# Data-Driven Design of Intelligent Wireless Networks: An Overview and Tutorial

**DOI:** 10.3390/s16060790

**Published:** 2016-06-01

**Authors:** Merima Kulin, Carolina Fortuna, Eli De Poorter, Dirk Deschrijver, Ingrid Moerman

**Affiliations:** Department of Information Technology, Ghent University-iMinds, Technologiepark-Zwijnaarde 15, Gent 9052, Belgium; carolina.fortuna@ijs.si (C.F.); eli.depoorter@intec.ugent.be (E.D.P.); dirk.deschrijver@intec.ugent.be (D.D.); ingrid.moerman@intec.ugent.be (I.M.)

**Keywords:** wireless networks, data science, data-driven research, machine learning, knowledge discovery, cognitive networking, intelligent systems

## Abstract

Data science or “data-driven research” is a research approach that uses real-life data to gain insight about the behavior of systems. It enables the analysis of small, simple as well as large and more complex systems in order to assess whether they function according to the intended design and as seen in simulation. Data science approaches have been successfully applied to analyze networked interactions in several research areas such as large-scale social networks, advanced business and healthcare processes. Wireless networks can exhibit unpredictable interactions between algorithms from multiple protocol layers, interactions between multiple devices, and hardware specific influences. These interactions can lead to a difference between real-world functioning and design time functioning. Data science methods can help to detect the actual behavior and possibly help to correct it. Data science is increasingly used in wireless research. To support data-driven research in wireless networks, this paper illustrates the step-by-step methodology that has to be applied to extract knowledge from raw data traces. To this end, the paper (i) clarifies when, why and how to use data science in wireless network research; (ii) provides a generic framework for applying data science in wireless networks; (iii) gives an overview of existing research papers that utilized data science approaches in wireless networks; (iv) illustrates the overall knowledge discovery process through an extensive example in which device types are identified based on their traffic patterns; (v) provides the reader the necessary datasets and scripts to go through the tutorial steps themselves.

## 1. Introduction

### 1.1. What Is Data Science?

Data science, also referred to as data-driven research, is research that puts a strong emphasis on starting from large datasets to solve a specific problem. The use of data science has gained popularity due to its capability to better understand the behavior of complex systems that cannot easily be modeled or simulated. For this reason, a possible definition of the term is provided by Dhar [1] and states that “data science is the study of the generalizable extraction of knowledge from data”. A simpler definition could be that data science enables discovering and extracting new knowledge from data. To this end, data-driven approaches start from datasets containing large amounts of experimental real-world data and utilize data science techniques to build statistical models that can be used to (i) better understand the behavior of the system and finally extract new knowledge; and (ii) generate synthetic data from the statistical model which mimics (simulates) the original observed data. Orders of magnitude larger than one are typically used in an analysis; often amounting to several gigabytes (possibly even terabytes) of traces. The use of data-driven research is increasing in various fields of science [2]. Example research areas in which data science was successfully applied include studying the human genome to predict the susceptibility of individual persons to specific diseases [3], analyzing connections in social networks [4], predicting the interests of customers based on previously purchased items [5], cloud computing applications [6,7], analysis of traffic in mobile cellular networks [8], *etc*.

Due to their unpredictable nature, wireless networks are an interesting application area for data science because they are influenced by both natural phenomena and man made artifacts. On one hand, they depend on electromagnetic propagation, which is a natural phenomenon, and on the other hand, they depend on the network technology consisting of hardware and software elements that were built by humans. Whereas well-defined aspects of wireless systems such as algorithm behavior, propagation on a specific type of channel, *etc*., can be modeled in simulations, the functioning of the overall systems is difficult to simulate. Due to these limitations, a number of wireless system behaviors cannot be identified and/or explained based on simulations alone. One such example is the fact that the inter-arrival time between wireless data packets depends on both protocol layer aspects and hardware behavior, a fact which will be exploited in this paper to identify devices and device types based on their traffic patterns. Other examples for the use of data science in wireless networks include the creation of complex system models, finding correlations and patterns between configurable parameters and network performance, predicting system behavior or identifying trade-offs such as Pareto fronts.

### 1.2. Motivation

Recently, an increasing number of research works in the wireless domain that rely on large datasets to prove their hypothesis can be seen [9,10,11,12]. While some of the "early adopters" of data-driven research such as [11] follow by the book the methodology used in the communities that developed data science without explicitly mentioning some domain specific terminology, other more recent works clearly use a data science approach [9,10]. By carefully studying example works, later analyzed in Section 3.7, that rely on large datasets, we noticed that, in some cases, the methodology used for solving the data science (data-driven) problem does not fully comply with the standard approaches (standard methodology) developed and accepted by the data science community. This can be explained by the difficulty of correctly grasping and understanding the knowledge discovery process, on which data science relies, by newcomers to the field. However, as a result, this may raise questions regarding the validity of some of the results.

Furthermore, related work such as [13,14,15,16] provide a comprehensive overview of generic data mining techniques and/or research studies that successfully applied those techniques to wireless sensor networks (WSN) and Internet of Things (IoT) networks. However, data mining is only a small step in the overall process of discovering knowledge from data. This means that just taking and using existing data mining algorithms, that seem best suited for a particular problem, is not always the most adequate approach to the overall problem. Algorithm selection, implementation and evaluation is possible and meaningful only after the problem is well defined and the data, including its statistical properties are analyzed and well understood. The formalization of the methodology for developing models based on empirical data in wireless networks is missing.

Similarly, related works such as [17] present an extensive survey of machine learning techniques, while [18] presents an extensive survey on computational intelligence algorithms (e.g., neural networks, evolutionary algorithms, swarm intelligence, reinforcement learning, *etc*.) that were used to address common issues in WSNs. Ref. [19] reviews various machine learning techniques that have been applied to cognitive radios (CR), while in [20] novel cooperative spectrum sensing algorithms for cognitive radio networks (CRN) based on machine learning techniques were evaluated. These research works ([13,14,15,16,17,18,19,20]) focus on existing application examples of different data science approaches to specific wireless networks. We encourage the reader to refer to the reference papers in this section to gain more insight on the algorithmic aspects of different data mining techniques and review a larger set of examples of data science applications to the wireless domain. Hence, by focusing on a application area, they narrow their context to the algorithms and approaches that are useful for that area rather than provide a generic methodology with the options and trade-offs available at each step.

Bulling *et al*. [21] present a comprehensive tutorial on human activity recognition (HAR) using on-body inertial sensors. Although, this work provides the breadth of information expected from an educational tutorial, it focuses on solving a particular data science problem (*i.e*., classification) for a particular application domain (*i.e*., HAR) with regard to the challenges of a particular wireless network scheme (*i.e*., body sensor networks). There is no single comprehensive tutorial on applying data science to various wireless networks, regardless on the application domain and type of problem that is to be solved, from an unified perspective (covering the overall process from the problem formulation, over data analysis, to learning and evaluation).

The aforementioned facts motivated the present tutorial paper and its ultimate goal to explain the knowledge discovery process underlying data science, and show how it can be correctly applied for solving wireless networking problems. This paper is provided at an opportune time, considering that:

- More and more data is generated by existing wireless deployments [22] and by the continuously growing network of everyday objects (the IoT).
- Data-driven research has found applications in various schemes of wireless networks [13], diverse fields of communication networks [6,7,8] and different fields of science in general [2].

Typically, wireless network researchers and experts are not (fully) aware of the potential that data science methods and algorithms can offer. Rather than having wireless network experts dive into data mining and machine learning literature, we provide the fundamental information in an easily accessible and understandable manner with examples of successful applications in the wireless domain. Hence, the main contribution of this paper is to offer a well-structured starting point for applying data science in wireless networks with the goal of minimizing the effort required from wireless professionals to start using data science methodology and tools to solve a domain specific problem. Ultimately, this knowledge will enable a better design of future wireless network systems such as the dense and heterogeneous wireless networks where humans, cars, homes, cities and factories will be monitored and controlled by such networked devices (IoT networks).

To the best of our knowledge, this is the first attempt to formally explain the correct approach and methodology for applying data science to the wireless domain. As such, we hope this tutorial paper will help bridge the gap and foster collaboration between system experts, wireless network researchers and data science researchers.

### 1.3. Contributions and Organization of the Paper

The paper aims to give a comprehensive tutorial on applying data science to the wireless domain by providing:

1. An overview of types of problems in wireless network research that can be addressed using data science methods together with state-of-the-art algorithms that can solve each problem type. In this way, we provide a guide for researchers to help them formulate their wireless networking problem as a data science problem.
2. A brief survey on the on-going research in the area of data-driven wireless network research that illustrates the diversity of problems that can be solved using data science techniques including references to these research works.
3. A generic framework as a guideline for researchers wanting to solve their wireless networking problem as a data science problem using best practices developed by the data science community.
4. A comprehensive hands-on introduction for newcomers to data-driven wireless network research, which illustrates how each component of the generic framework can be instantiated for a specific wireless network problem. We demonstrate how to correctly apply the proposed methodology by solving a timely problem on fingerprinting wireless devices, that was originally introduced in [12]. Finally, we show benefits of using the proposed framework compared to taking a custom approach.
5. The necessary scripts to instantiate the proposed framework for the selected wireless networking problem [23], complemented with a publicly available datasets [24].

According to the aforementioned, the remainder of this paper is organized as follows: Section 2 elaborates the use of data science in wireless networks. Section 3 introduces a generic framework for applying the correct methodology for data-driven wireless network research. Section 4 details how each component of the framework can be executed in a time efficient manner using best practice developed by the data science community. This process is extensively illustrated by a case study that demonstrates how the proposed framework can be implemented to solve a wireless networks classification problem. We underline the significance of the correct methodology by comparing the proposed solution against an existing work presented in [12]. Section 5 concludes the paper.

## 2. Introduction to Data Science in Wireless Networks

This section introduces the basic terminology used in data science in order to set up the necessary fundamentals for the reader, and examines the applicability of recent advances in data science to the wireless networking domain. Thus, this section (i) introduces the basic concepts of learning and different learning paradigms used in data science; (ii) describes which categories of problems in wireless networks can be answered by data science approaches; (iii) describes a number of popular data science algorithms that can solve these categories of problems and state-of-the-art achievements in applying these algorithms to various wireless network use cases.

With this in regard, this section is both, a brief survey on existing work in data-driven wireless research and a starting guide for researchers wanting to apply data science to problems related to wireless networks.

### 2.1. Types of Learning Paradigms

The ultimate goal of data science is to extract knowledge from data, *i.e*., turn data into real value [25]. At the heart of this process are severe algorithms that can learn from and make predictions on data. As such, these algorithms are referred to as learning algorithms and are part of the machine learning and data mining fields of study (the differences between the two fields are detailed in Section 2.1.1).

In the context of wireless networks, learning is a mechanism that enables context awareness and intelligence capabilities in different aspects of wireless communication. Over the last years, it has gained popularity due to its success in enhancing network-wide performance (*i.e*., the Quality of Service, QoS) [26], facilitating intelligent behavior by adapting to complex and dynamically changing (wireless) environments [27] and its ability to add automation for realizing concepts of self-healing and self-optimization [28]. During the past years, different learning approaches have been applied in various wireless networks schemes such as medium access control [29,30], routing [9,10], data aggregation and clustering [31,32], localization [33,34], energy harvesting communication [35], cognitive radio [36,37], *etc*. These schemes apply to a variety of wireless networks such as: mobile ad hoc networks [38], wireless sensor networks [18], wireless body area networks [39], cognitive radio networks [20,40] and cellular networks [41].

#### 2.1.1. Data Mining *vs*. Machine Learning

In the literature, machine learning and data mining are terms used interchangeably. It is difficult to make a clear difference between the two, as they appear in the same context and very often rely on the same algorithms and techniques (e.g., decision trees, logistic regression, neural networks, *etc*.). Perhaps the best way to explain the difference is by looking at their scope.

Data mining aims to discover new, previously unseen knowledge in large datasets. It focuses on helping humans understand complex relationships between data. For instance, it enables marketing experts to segment customers based on previous shopping habits. Another example is applying learning algorithms to extract the shopping patterns of thousands of individuals over time; then being presented with a new individual, having a very short shopping history (e.g., five items), the learned model is able to automatically tell to which of the segments discovered by the data mining process the new individual belongs. Hence, data mining tends to be focused on solving actual problems encountered in practice by exploiting algorithms developed by the machine learning community.

Machine learning on the other hand, aims to develop algorithms and techniques that give computers the ability to learn to recognize information or knowledge (*i.e*., patterns) automatically without being explicitly programmed to do so. This is why machine learning algorithms are typically referred to as learning algorithms. Machine learning experts focus on proving mathematical properties of new algorithms, while data mining experts focus on understanding empirical properties of existing algorithms that they apply.

#### 2.1.2. Supervised *vs.* Unsupervised *vs.* Semi-Supervised Learning

Learning can be categorized by the amount of knowledge or feedback that is given to the learner as either supervised or unsupervised.

##### Supervised Learning

Supervised learning utilizes predefined inputs and known outputs to build a system model. The set of inputs and outputs forms the labeled training dataset that is used to teach a learning algorithm how to predict future outputs for new inputs that were not part of the training set. Supervised learning algorithms are suitable for wireless network problems where prior knowledge about the environment exists and data can be labeled. For example, predict the location of a mobile node using an algorithm that is trained on signal propagation characteristics (inputs) at known locations (outputs). Various challenges in wireless networks have been addressed using supervised learning such as: medium access control [29,42,43,44], routing [45], link quality estimation [46,47], node clustering in WSN [48], localization [49,50,51], adding reasoning capabilities for cognitive radios [36,37,52,53,54,55,56], *etc*. Supervised learning has also been extensively applied to different types of wireless networks application such as: human activity recognition [21,39,57,58,59,60], event detection [61,62,63,64,65], electricity load monitoring [66,67], security [68,69,70], *etc*. Some of these works will be analyzed in more detail later.

##### Unsupervised Learning

In contrast, unsupervised learning algorithms try to find hidden structures in unlabeled data. The learner is provided only with inputs without known outputs, while learning is performed by finding similarities in the input data. As such, these algorithms are suitable for wireless network problems where no prior knowledge about the outcomes exists, or annotating data (labelling) is difficult to realize in practice. For instance, automatic grouping of wireless sensor nodes into clusters based on their current sensed data values and geographical proximity (without knowing a priori the group membership of each node) can be solved using unsupervised learning. In the context of wireless networks, unsupervised learning algorithms are widely used for: data aggregation [31], node clustering for WSNs [31,71,72,73], data clustering [74,75,76], event detection [77] and several cognitive radio applications [78,79].

##### Semi-Supervised Learning

Several mixes between the two learning methods exist and materialize into semi-supervised learning [80]. Semi-supervised learning is used in situations when a small amount of labeled data with a large amount of unlabeled data exists. It has great practical value because it may alleviate the cost of rendering a fully labeled training set, especially in situations where it is infeasible to label all instances. For instance, in human activity recognition systems where the activities change very fast so that some activities stay unlabeled or the user is not willing to cooperate in the data collection process, supervised learning might be the best candidate to train a recognition model [81,82,83]. Other potential use cases in wireless networks might be localization systems where it can alleviate the tedious and time-consuming process of collecting training data (calibration) in fingerprinting-based solutions [84] or semi-supervised traffic classification [85], *etc*.

#### 2.1.3. Offline *vs.* Online *vs.* Active Learning

Learning can be categorized depending on the way the information is given to the learner as either offline or online learning. In offline learning the learner is trained on the entire training data at once, while in online learning the training data becomes available in a sequential order and is used to update the representation of the learner in each iteration.

##### Offline Learning

Offline learning is used when the system that is being modeled does not change its properties dynamically. Offline learned models are easy to implement because the models do not have to keep on learning constantly, and they can be easily retrained and redeployed in production. For example, in [9] a learning-based link quality estimator is implemented by deploying an offline trained model into the network stack of Tmote Sky wireless nodes. The model is trained based on measurements about the current status of the wireless channel that are obtained from extensive experiment setups from a wireless testbed.

Another use cases are human activity recognition systems, where an offline trained classifier is deployed to recognize actions from users. The classifier model can be trained based on information extracted from raw measurements collected by sensors integrated in a smartphone, which is at the same time the central processing unit that implements the offline learned model for online activity recognition [86].

##### Online Learning

Online learning is useful for problems where training examples arrive one at a time or when due to limited resources it is computationally infeasible to train over the entire dataset. For instance, in [87] a decentralized learning approach for anomaly detection in wireless sensor networks is proposed. The authors concentrate on detection methods that can be applied online (*i.e*., without the need of an offline learning phase) and that are characterized by a limited computational footprint, so as to accommodate the stringent hardware limitations of WSN nodes.

Another example can be found in [88], where the authors propose an online outlier detection technique that can sequentially update the model and detect measurements that do not conform to the normal behavioral pattern of the sensed data, while maintaining the resource consumption of the network to a minimum.

##### Active Learning

A special form of online learning is active learning where the learner first reasons about which examples would be most useful for training (taking as few examples as possible) and then collects those examples. Active learning has proven to be useful in situations when it is expensive to obtain samples from all variables of interest. Recently, the authors in [89] proposed a novel active learning approach (for graphical model selection problems), where the goal is to optimize the total number of scalar samples obtained by allowing the collection of samples from only subsets of the variables. This technique could for instance alleviate the need for synchronizing a large number of sensors to obtain samples from all the variables involved simultaneously.

Active learning has been a major topic in recent years in machine learning and an exhaustive literature survey is beyond the scope of this paper. We refer the reader to [90,91,92] for more details on state-of-the-art and progress on active learning algorithms.

Table 1 summarizes the previously introduced learning paradigms.

### 2.2. Types of Data Science Problems in Wireless Networks

As shown in previous section, data science has been successfully applied in different areas of wireless networks. Prior to applying data science techniques to any wireless network problem, the problem has to be first translated into an adequate data mining method. This section guides the reader on how to formulate the wireless networking problem as a data science problem, making a first step towards the broader knowledge discovery process that will be formalized in Section 3. For each type of problem, the most popular learning algorithms and their relation to the previously introduced learning paradigms is identified.

#### 2.2.1. Regression

Regression is a data mining method that is suitable for problems that aim to predict a real-valued output variable. It is a supervised learning method, which models (*i.e*., fits) a set of known inputs (*i.e*., explanatory or independent variables) and corresponding outputs (*i.e*., dependent variable) with the most suitable mathematical representation (*i.e*., function or model). Depending on the function representation, regression techniques can be categorized into linear and non-linear regression algorithms.

##### Linear Regression

Linear regression is a technique for modeling the relationship between the input (*x*) and output variable (*y*) so that the output is a linear combination of the input variables (dependent variable).

(1) $$y\left(x\right)=\theta_{0}+\theta_{1}x_{1}+...+\theta_{n}x_{n}=\theta_{0}+\sum_{i=1}^{n}\theta_{i}x_{i}$$

where $x={\left(x_{1},...x_{n}\right)}^{T}$.

A simple example is linear regression with one input variable (*i.e*., univariate linear regression), which fits the data (inputs *x* and predictions *y*) with a linear function, e.g., f(x)=ax+b. This function, f(x), is supposed to predict future values, f(x), based on new inputs (*i.e*., *x*).

***Linear regression use case in wireless networks***. In the context of wireless networks, linear regression is frequently used to derive an empirical log-distance model for the radio propagation characteristics as a linear mathematical relationship between the Received Signal Strength Indication (RSSI), usually in dBm, and the distance. This model can be used in RSSI-based indoor localization algorithms to estimate the distance towards each fixed node (*i.e*., anchor node) in the ranging phase of the algorithm [33].

##### Nonlinear Regression

Nonlinear regression is a regression method which models the observed data by a function that is a nonlinear combination of the model parameters and one or more independent variables.

***Nonlinear regression use case in wireless networks***. For instance, in [93] non-linear regression is used to model the relationship between the SINR (Signal to Interference plus Noise Ratio) and PRR (Packet Reception Rate) that could improve the design and analysis of higher layer protocols. Similarly, non-linear regression techniques are extensively used for modeling the relation between the PRR and the RSSI, as well as between PRR and the Link Quality Indicator (LQI), to build a mechanism to estimate the link quality based on observations (RSSI, LQI) [94].

#### 2.2.2. Classification

A classification problem tries to understand and predict discrete values or categories while a regression problem targets continuous valued problems. The term classification comes from the fact that it predicts the class membership of a particular input instance. Classification problems can be solved by supervised learning approaches, that aim to model boundaries between sets (*i.e*., classes) of similar behaving instances, based on known and labeled (*i.e*., with defined class membership) input values. The learned model is used to map future input instances (X) to a particular class (Y). A detailed example will be given in Section 4 where devices and device types are classified based on the packet inter-arrival times from a publicly available dataset from wireless devices. Similarly, identifying the application layer protocol of a traffic flow can be solved as a classification problem: traffic classification based on the statistical properties of traffic traces [11]. There are many learning algorithms that can be used to classify data including decision trees, k-nearest neighbours, logistic regression, support vector machines and neural networks.

##### Neural Networks

Neural Networks (NN) [95] or artificial neural networks (ANN) is a supervised learning algorithm inspired on the working of the brain, that is typically used to derive complex, non-linear decision boundaries for building a classification model, but are also suitable for training regression models when the goal is to predict real-valued outputs (regression problems are explained in Section 2.2.1). Neural networks are known for their ability to identify complex trends and detect complex non-linear relationships among the input variables at the cost of higher computational burden. A neural network model consists of one input, a number of hidden layers and one output layer. The input layer corresponds to the input data variables. Each hidden layer consists of a number of processing elements called neurons that process its inputs (the data from the previous layer) using an activation or transfer function that translates the input signals to an output signal. Commonly used activation functions are: unit step function, linear function, sigmoid function and the hyperbolic tangent function. The elements between each layer are highly connected by connections that have numeric weights that are learned by the algorithm. The output layer outputs the prediction (*i.e*., the class) for the given inputs and according to the interconnection weights defined through the hidden layer. The algorithm is again gaining popularity in recent years because of new techniques and more powerful hardware that enable training complex models for solving complex tasks. In general, neural networks are said to be able to approximate any function of interest when tuned well, which is why they are considered as universal approximators [96].

***Neural networks use case in wireless networks***. In [97], the authors proposed a neural network based mechanism for dynamic channel selection in an IEEE 802.11 network. The neural network is trained to identify how environmental measurements and the status of the network affect the performance experienced on different channels. Based on this information it is possible to dynamically select the channel which is expected to yield the best performance for the mobile users.

##### Deep Learning

Recently, it has been noticed that the same amount of complexity as modeled by a neural networks with one hidden layer and several neurons can be gained with multiple hidden layers that have less neurons in total. Such networks are known as deep neural networks and the learning process is known as deep learning. Deep neural networks hold the potential to replace the process of manually extracting features, which depends much on prior knowledge and domain expertise, with unsupervised or semi-supervised feature learning techniques [98]. Various deep learning techniques such as deep neural networks (DNN), convolutional neural networks (CNN), recurrent neural networks (RNN) and deep belief networks (DBN) have shown success in various fields of science including computer vision, automatic speech recognition, natural language processing, bioinformatics, *etc*. Although deep networks showed excellent performance in many challenging machine learning tasks, their application to wireless networks has not yet been widely explored. We present two recent advances applying deep learning techniques to the wireless domain [99,100].

***Deep learning use case in wireless networks***. In [99], deep neural networks (DNN) have been applied to wireless sensor networks. The authors proposed a distributed learning model by dividing the deep neural network into different layers and deploying them on several sensor nodes. The proposed solution aims to reduce power consumption in WSNs by reducing the number of data that has to be transmitted, by transmitting only data that is processed by the DNN layer locally at the node instead of the full raw data.

In [100] a new intelligent communication systems is proposed called Cognition-Based Networks (COBANETS) whose main building blocks are (i) unsupervised deep learning networks as the enabler for learning, modeling and optimization of networks, and (ii) software defined networking (SDN) as the enabler for reconfiguration of the network protocol stack and flexible network management, making it possible to actuate network-wide optimization strategies.

##### Decision Trees

Decision trees [101] is a supervised learning algorithm that creates a tree-like graph or model that represents the possible outcomes or consequences of using certain input values. The tree consists of one root node, internal nodes called decision nodes which test its input against a learned expression, and leaf nodes which correspond to a final class or decision. The learning tree can be used to derive simple decision rules that can be used for decision problems or for classifying future instances by starting at the root node and moving through the tree until a leaf node is reached where a class label is assigned. However, decision trees can achieve high accuracy only if the data is linearly separable, *i.e*., if there exists a linear hyperplane between the classes. Hence, constructing an optimal decision tree is NP-complete [102].

There are many algorithms that can form a learning tree such as the simple Iterative Dichotomiser 3 (ID3), its improved version C4.5, *etc*.

***Decision trees use case in wireless networks***. We consider the problem of designing an adaptive MAC layer as an application example of decision trees in wireless networks. In [29] a self-adapting MAC layer is proposed. It is composed of two parts: (i) a reconfigurable MAC architecture that can switch between different MAC protocols at run time, and (ii) a trained MAC engine that selects the most suitable MAC protocol for the current network condition and application requirements. The MAC engine is solved as a classification problem using a decision tree classifier which is learned based on: (i) two types of input variables which are (1) network conditions reflected through the RSSI statistics (*i.e*., mean and variance), and (2) the current traffic pattern monitored through the Inter-Packet Interval statistics (*i.e*., mean and variance) and application requirements (*i.e*., reliability, energy consumption and latency), and (ii) the output which is the MAC protocol that is to be predicted and selected.

##### Logistic Regression

Logistic regression [103] is a simple supervised learning algorithm widely used for implementing linear classification models, meaning that the models define smooth linear decision boundaries between different classes. At the core of the learning algorithm is the logistic function which is used to learn the model and predict future instances.

***Logistic regression use case in wireless networks***. Liu *et al*. [9] improved multi-hop wireless routing by creating a data-driven learning-based radio link quality estimator. They investigated whether machine learning algorithms (e.g., logistic regression, neural networks) can perform better than traditional, manually-constructed, pre-defined estimators such as STLE (Short-Term Link Estimator) [104] and 4Bit (Four-Bit) [105]. Finally, they selected logistic regression as the most promising model for solving the following classification problem: predict whether the next packet will be successfully received, *i.e*., output class is 1, or lost, *i.e*., output class is 0, based on the current wireless channel conditions reflected by statistics of the PRR, RSSI, SNR and LQI.

While in [9] the authors used offline learning to do prediction, in their follow-up work [10], they went a step further and both training and prediction were performed online by the nodes themselves using logistic regression with online learning (more specifically the stochastic gradient descent online learning algorithm). The advantage of this approach is that the learning and thus the model, adapt to changes in the wireless channel, that could otherwise be captured only by re-training the model offline and updating the implementation on the node.

##### SVM

Support Vector Machine (SVM) [106] is a learning algorithm that solves classification problems by first mapping the input data into a higher-dimensional feature space in which it becomes linearly separable by a hyperplane, which is used for classification. The mapping from the input space to the high-dimensional feature space is non-linear, which is achieved using *kernel* functions. Different kernel functions comply best for different application domains. There are three types of popular kernel functions: linear kernel, polynomial kernel and Gaussian radial basis kernel function (RBF).

***SVM use case in wireless networks***. SVMs have been extensively used in cognitive radio applications to perform signal classification. For this purpose, typically flexible and reconfigurable SDR (software defined radio) platforms are used to sense the environment to obtain information about the wireless channel conditions and users’ requirements, while intelligent algorithms build the cognitive learning engine that can make decisions on those reconfigurable parameters on SDR (e.g., carrier frequency, transmission power, modulation scheme).

In [36,37,107] SVMs are used as the machine learning algorithm to classify signals among a given set of possible modulation schemes. For instance, Huang *et al*. [37] identified four spectral correlation features that can be extracted from signals for distinction of different modulation types. Their trained SVM classifier was able to distinguish six modulation types with high accuracy: AM, ASK, FSK, PSK, MSK and QPSK.

##### k-NN

*k* nearest neighbors (k-NN) [108] is a learning algorithm that can solve classification and regression problems by looking into the distance (closeness) between input instances. It is called a non-parametric learning algorithm because, unlike other supervised learning algorithms, it does not learn an explicit model function from the training data. Instead, the algorithm simply memorizes all previous instances and then predicts the output by first searching the training set for the *k* closest instances and then: (i) for classification-predicts the majority class amongst those *k* nearest neighbors, while (ii) for regression-predicts the output value as the average of the values of its *k* nearest neighbors. Because of this approach, k-NN is considered a form of instance-based or memory-based learning.

In the context of this section, k-NN will be exemplified for solving a classification wireless problem. k-NN is widely used since it is one of the simplest forms of learning. It is also considered as *lazy* learning as the classifier is passive until a prediction has to be performed, hence no computation is required until performing classification.

***k-NN use case in wireless networks***. In [109] the goal of determining the activity of a human who is wearing attached sensor nodes is turned into a classification problem. The sensor nodes are capturing and recording acceleration data, which is then sent via Bluetooth to the classifier. In [109], k-NN was one of the candidates to solve the classification problem. Acceleration data (x, y, and z coordinates) gathered from acceleration sensors is transformed into input data such as: step count, area between mean of local maxima and signal, crossing of mean value, mean value of local maxima and the angle of each axis in relation to the gravity, and fed into the classifier. k-NN calculates the distance between each input instance to be classified and all the remaining training instances. Classification is performed according to how many instances of a certain class are nearest.

#### 2.2.3. Clustering

Clustering is a data mining method that can be used for problems where the goal is to group sets of similar instances into clusters. Opposed to classification, it uses *unsupervised* learning, which means that the input dataset instances used for training are not labeled, *i.e*., it is unknown to which group they belong. The clusters are determined by inspecting the data structure and grouping objects that are similar according to some metric. Clustering algorithms are widely adopted in wireless sensor networks, where they have found use for grouping sensor nodes into clusters to satisfy scalability and energy efficiency objectives, and finally elect the head of each cluster. Recently, a large number of node clustering algorithms have been proposed for WSNs [110]. However, these *node* clustering algorithms typically do not use the data science clustering techniques directly. Instead, they exploit *data* clustering techniques to find data correlations or similarities between data of neighboring nodes, that can be used to partition sensor nodes into clusters.

Clustering can be used to solve other types of problems in wireless networks like anomaly detection, *i.e*., outliers detection, such as intrusion detection or event detection, for different data pre-processing tasks (data pre-processing is detailed in Section 3.3), cognitive radio application (e.g., identifying wireless systems [79]), *etc*. There are many learning algorithms that can be used for clustering, but the most commonly used is *k*-Means.

##### *k*-Means

*k*-Means is an unsupervised learning clustering algorithm that simply partitions the input data instances into *k* clusters, so that the resulting intra-cluster similarity is high, while the inter-cluster similarity low. The similarity is measured with respect to the mean value of the instances in a cluster.

***k-Means use case in wireless networks***. In [74] a distributed version of the *k*-Means clustering algorithm was proposed for clustering data sensed by sensor nodes. The clustered data is summarized and sent towards a sink node. Summarizing the data ensures to reduce the communication transmission, processing time and power consumption of the sensor nodes.

Other popular clustering algorithms include hierarchical clustering methods such as single-linkage, complete-linkage, centroid-linkage; graph theory-based clustering such as highly connected subgraphs (HCS), cluster affinity search technique (CAST); kernel-based clustering as is support vector clustering (SVC), *etc*. A novel two-level clustering algorithm, namely TW-*k*-means, has been introduced by Chen *et al*. [32]. For a more exhaustive list of clustering algorithms and their explanation we refer the reader to [111].

#### 2.2.4. Anomaly Detection

Anomaly detection (changes and deviation detection) is used when the goal is to identify unusual, unexpected or abnormal system behavior. This type of problem can be solved by supervised or unsupervised learning depending on the amount of knowledge present in the data (*i.e*., whether it is labeled or unlabeled, respectively). Accordingly, classification and clustering algorithms can be used to solve anomaly detection problems. A wireless example is the detection of suddenly occurring phenomena, such as the identification of suddenly disconnected networks due to interference or incorrect transmission power settings. It is also widely used for outliers detection in the pre-processing phase [112]. Other use-case examples include intrusion detection, fraud detection, event detection in sensor networks, *etc*.

***Anomaly Detection use case in wireless networks***. We consider an anomaly detection use case in the context of WSN security. Namely, WSNs have been target of many types of DoS attacks. The goal of DoS attacks in WSNs is to transmit as many packets as possible whenever the medium is detected to be idle. This prevents a legitimate sensor node from transmitting their own packets. To combat a DoS attack, a secure MAC protocol based on neural networks has been proposed in [42]. The NN model is trained to detect an attack by monitoring variations of following parameters: collision rate $R_{c}$, average waiting time of a packet in MAC buffer $T_{w}$, arrival rate of RTS packets $R_{RTS}$. An anomaly, *i.e*., attack, is identified when the monitored traffic variations exceeds a preset threshold, after which the WSN node is switched off temporarily. The results is that flooding the network with untrustworthy data is prevented by blocking only affected sensor nodes.

In [87] online learning techniques have been used to incrementally train a neural network for in-node anomaly detection in wireless sensor network. More specifically, the Extreme Learning Machine algorithm [113] has been used to implement classifiers that are trained online on resource-constrained sensor nodes for detecting anomalies such as: system failures, intrusion, or unanticipated behavior of the environment.

#### 2.2.5. Summarization

Summarization is used in problems where the goal is to find a compact (summarized) representation of data. The method typically utilizes different summary statistics to find a reduced representation of the data. It is frequently used in data analysis, data pre-processing, data visualization and automated report generation tasks. Accordingly, this method can be rather seen as an optimization problem with the compaction gain and information loss as objective functions, whereas it is not suitable to solve a prediction problem. As such, summarization does not conform to any of the learning paradigms previously introduced, hence is not meant to solve a knowledge discovery problem. However, summarization techniques have been heavily used to address challenges that are crucial for wireless communication systems and they can be realized by some of the aforementioned learning-based data science methods. Thus, it is important to give a short overview of potential summarization techniques used in wireless networks. First, we would like to point the reader to some of the recently emerging non-learning-based summarization techniques that are adopted in wireless networks including compressive sensing, expectation-maximization, non-negative matrix factorization, *etc*.

Compressive sensing (CS) has shown great success in conjunction with principal component analysis for the design of efficient data aggregation schemes in wireless networks. In short, it replaces the traditional “sample then compress” summarization scheme with “sample while compressing”. We refer the reader to [114] for more details on compressive sensing techniques. Some example works that integrate compressive sensing techniques for reducing data transmission can be found in [115,116], while [117] exploits CS for energy efficient vital signal telemonitoring in wireless body area networks.

Expectation-maximization (EM) is an iterative method composed of two steps (i) expectation (E) where a cost function is defined while fixing the current expectation of the system parameters, and (ii) maximization (M) where the parameters are recomputed so that the cost function is minimized. EM is typically used in combination with principal component analysis to enhance data aggregation schemes in wireless networks.

Non-negative matrix factorization is a method for factorizing a matrix into two new matrices with the property that all three matrices do not contain negative elements. In practice, this non-negativity makes it easier to inspect matrices and has found many applications for designing clustering algorithms and signal processing tasks.

Similarly as anomaly detection summarization can be realized by some of the previously introduced methods, *i.e*., learning-based schemes, which is the focus of this paper. For example, several clustering approaches have shown promise for designing efficient data aggregation.

***Summarization use case in wireless networks***. Data summarization techniques are typically used to design more efficient communication strategies in low power wireless sensor networks constrained. Given the fact that the most of the energy on the sensor nodes is consumed while the radio is turned on, *i.e*., while sending and receiving data [118], data aggregation techniques can be used to reduce transmission and hence energy consumption. In [31] a data aggregation scheme is proposed for in-network data summarization to save energy and reduce computation in wireless sensor nodes. The proposed algorithm first uses clustering as a method to form clusters of nodes sensing similar values within a given threshold, and then only one sensor reading per cluster is transmitted which lowered extremely the number of transmissions in the wireless sensor network.

### 2.3. Summary

Although the main goal of all data science methods is to find useful information hidden in the data, previous section showed that the original objectives are different. For example, classification is used to classify a set of patterns whereas regression is used to predict continuous outcomes. Thus, it is important to select the correct method for the target wireless networking problem. Several researches showed that the presented methods have the potential to extract useful knowledge that can be exploited to solve a wide variety of problems in the wireless domain.

Table 2 presents a brief overview of the types of wireless problems that can be addressed with data science techniques with regard to the application domain, including references to example applications that were introduced in Section 2.2.

As it can be seen, data science methods can be applied to different facets of wireless networks problems, that depending on the goal typically fall into one of the two categories: enhancing network performance and information processing for different wireless applications. A promising trend that can be found from these examples is that data science is able to optimize different aspects of wireless networks or make even more intelligent.

## 3. A Generic Framework for Applying Data Science in Wireless Networks

There is a well established process used already for decades, that enables discovering new knowledge in large datasets—it is referred to as the Knowledge Discovery (KD) process [119]. There are many variations of the knowledge discovery process. Some authors describe a 9-step process, others a 5- or 6-step process; however, the differences in opinion typically happen at the data pre-processing step which is seen as one step with four sub-steps by some authors and as four different steps by others [120].

**Figure 1:**
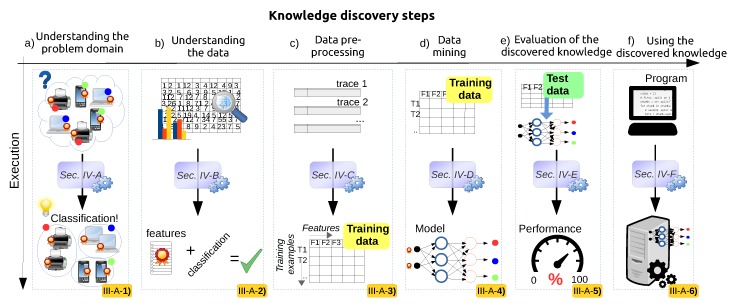
Transformations through each step of the data-driven knowledge discovery process: (**a**) Understanding the problem domain; (**b**) Understanding the data; (**c**) Data pre-processing; (**d**) Data mining; (**e**) Evaluation of the discovered knowledge; (**f**) Using the discovered knowledge.

Inspired by this process, this paper formalizes a six-step knowledge discovery framework for applying data science to different aspects of wireless networks. Figure 1 depicts these six steps on the horizontal axis, while the vertical axis summarizes the transformations happening within each step.

The focus of this section is to explain what is done in each step, in terms of its goals and the final output/results, as well as the type of inputs that a step expects from the previous one. Afterwards, Section 4 further elaborates each step from a hands-on perspective, *i.e*., demonstrates how these steps should be carried out and which are the challenges, decisions and trade-offs we are faced with at each one of them.

### 3.1. Understanding the Problem Domain

The goal of this step is to clearly identify and state the wireless networking problem that needs to be solved and formulate it as a data science problem. The formulation has to explicitly and clearly describe how data can be used to find answers. For instance, it has to be defined if it is a classification or a regression problem (see Section 2.2). This step must also identify the collection requirements for the data (*i.e*., which parameters to collect, for how long, *etc*.). Typically, a team of experts in different domains collaborates in this step including statisticians, computer scientists working in the domain of data mining and machine learning and domain experts. As a results the problem is elaborated and translated into data mining goals, and an initial data definition and selection of data mining tools to be used later in the process is performed.

As an example, consider the following wireless networking problem [9]. Liu *et al*. [9] identified the following wireless networking problem. Reliable multi-hop routing in wireless networks can be improved by utilizing a metric that optimally describes the wireless link behavior. To this end, the authors proposed collected and analyzed a large amount of link layer data, namely the Packet Reception Rate (PRR), and physical layer data, namely the Received Signal Strength Indicator (RSSI), Signal to Noise Ratio (SNR) and Link Quality Indicator (LQI). Then, the authors used a window mean estimator with exponentially weighted moving average (WMEWMA) as well as the following machine learning algorithms to predict the link quality: Bayes classifier [121], logistic regression and artificial neural networks (ANN).

### 3.2. Understanding the Data

The goal of this step is to collect the data according to the data definition stated in the previous step, and explore the collected data in order to validate both the dataset and the formulation of the data science problem from the previous step. In this way, the researchers can assess (i) whether the selected data is a representative sample for solving the formulated problem, and (ii) whether the stated hypothesis is true and the selected data mining task is likely to prove it. For this purpose, data science experts often use simple visual representations of data, some simple statistics (*i.e*., avg, standard deviation, min, max, quartiles, *etc*.) and perhaps also some domain-specific metrics such as the coefficient of determination $R^{2}$, coefficient of correlation (Pearson’s product moment correlation coefficient), *etc*. This helps them see whether one value, also referred to as the target value, label or class can be predicted from or is in some way determined by the other available values, also referred to as dependent values, features or arguments. At the end, this step should indicate whether the researcher can further proceed with the KD framework, *i.e*., with pre-processing the data, or should iterate back to the first step to revise the problem definition and/or collect new data.

To reuse the previous example, [9] states that the physical layer parameters (RSSI, SNR, LQI) are measurements that reflect the quality of the wireless channel. To test their hypothesis, they plotted the PRR variation (estimation for the link quality) as a function of RSSI, SNR and LQI variation, respectively. Their results showed obvious correlations between the wireless link quality and the physical layer data, and gave the indication that it is possible to model the dependency between those variables by simply plotting the data (*i.e*., PRR=f(RSSI), PRR=f(SNR), PRR=f(LQI)). In this work, the values of the coefficient of determination $R^{2}$ for SNR and LQI were 0.87 and 0.93, respectively. Obtaining high $R^{2}$ values is often an indication of significant correlation with PRR. RSSI had a much lower $R^{2}$ value of 0.43. These results suggest that it is possible to leverage the SNR and LQI measurements to predict link quality with relatively high accuracy, while RSSI can only be used to a lesser extent. Hence, physical layer data could be used as a good input for the prediction process of expected PRR.

### 3.3. Data Pre-Processing

The goal of this step is to transform the collected data, also referred to as raw data, into a format suitable for feeding into data mining and machine learning algorithms, also referred to as training data. The raw data may be unsuitable for direct use by the algorithms for several reasons, such as:

- raw data often contains values that do not reflect the real behavior of the target problem (e.g., faulty measurements);
- data is spread over multiple sources (e.g., across several databases);
- data does not have the most optimal form for efficient training (e.g., parameters with different scales);
- data contains irrelevant or insignificant measurements/parameters (e.g., a system parameter that is not likely to help solve the problem).

The term pre-processing emphasizes that the data has to be processed prior to training. The sub-steps used by the KD framework to transform the data from raw to training data are: data cleaning, integration, reduction and transformation.

**Data cleaning** is the step where abnormal observation points (*i.e*., measurements) are detected, and then corrected or removed from the dataset. Those observations are referred to as outliers. Various outlier detection techniques can be used at this stage. For instance, in [9] the PRR plotted as a function of a physical layer parameter can be a graphical method of detecting outliers. The data plot for the LQI prediction parameter PRR=f(LQI) shows few points that are very distant from the remaining dataset. One reason for their occurrence comes from the fact that data derived from sensors is prone to measurement errors. Removing these values from the dataset can lead to a new more accurate prediction model.

**Data integration** is required when using multiple databases or files. This sub-step applies to larger systems where objects are stored in (multiple) databases which often happens in complex web based production systems, however it is less likely to be encountered in most problems concerning wireless networks.

**Data transformation** implies finding a new form of the data that is more suitable to train the mining/learning algorithms. This process typically performs normalization. It has to be noted that the term *normalization* that is used in data pre-processing has also other connotations in statistics. on the input *features* by scaling them so as to fall within a smaller range. For instance, in [9] the physical layer parameters (RSSI, SNR and LQI) range in the intervals: [55,45], [0,50] and [40,110], respectively, while the PRR values fall within the range [0,1]. Bringing the system parameters to the same scale by normalizing the physical layer parameters to [0,1] will often computationally speed-up the learning and prediction process the algorithms.

**Data reduction** is about removing irrelevant information with respect to the goal of the task. In other words, we need to make sure that only relevant instances and system properties are selected for training. For this purpose different feature selection (finding useful features to represent the data) and extraction algorithms that reduce the datasets dimensionality are used. For instance, RSSI has been shown in [9] to have a poor fit for the PRR function, only the SNR and LQI could be selected as representative features to form the vector used as input for the link quality prediction model.

After completing the four sub-steps, the data will be available in a format suitable for training, often referred to as feature vectors. The feature vectors are a mathematical representation upon which calculations can be performed. For instance, one feature vector could be a collection of different wireless network parameters such as RSSI and SNR. In this case the feature vector would be ${\left[RSSI,SNR\right]}^{T}$. There is an entire research branch concerned with feature engineering [122], however, such details are beyond the scope of this paper.

### 3.4. Data Mining

The goal of this step is to train the data mining/machine learning algorithms to solve the KD problem that was identified and formulated in step 1.

As elaborated in Section 2.1.2 , algorithms can be classified into two major subgroups: supervised and unsupervised. Supervised methods require two input types: the feature vector and the label (or target) vector for training. The label vector represents the true class or value corresponding to the feature vector and has been determined during the data collection process. The label vector is the target variable that needs to be predicted for future measurements. Each feature vector together with its corresponding label vector from the training set represents a training example. Hence, training examples are vectors in a multidimensional feature space with corresponding labels. In contrast, unsupervised methods do not use labels and are more suitable for finding structure in unlabeled data or for predicting the value of continuous parameters based on the feature vectors.

There is a trade-off between the length of the feature vector, the computational speed of the algorithm and the accuracy of the prediction. The algorithm is supposed to build a model by calculating the model coefficients, based on pairs of known features and corresponding known predictions (*i.e*., the training set). Finding the optimal algorithm (see Section 2.2 for some examples) typically requires more runs and iterations than the previous and next steps since each algorithm has a number of tunable parameters and the goal is to find the best performing configuration.

For instance, in the previously discussed example from [9] there are several combinations of input feature vectors that are used: ${\left[PRR_{i},RSSI_{i},SNR_{i},LQI_{i}\right]}^{T}$, ${\left[PRR_{i},RSSI_{i}\right]}^{T}$, ${\left[PRR_{i},SNR_{i}\right]}^{T}$, *etc*. The label vector is binary, having the value 1 for successfully received packets and 0 for lost packets. Additional system parameters that are tuned during this step are (1) the window size on which a prediction is made, *W* (*i.e*., predict whether the next packet will be received based on the last 1, 10, 20, *etc*. packets), (2) the number of the considered links, *L* (*i.e*., all links in the network, a subset or a single link), (3) the number of packets used for generating the dataset, *P*, (4) the packet inter-arrival time, *I*, which models different traffic behaviours and (5) model specific parameters such as the number and size of the hidden layers of the neural network. Altogether, in [9], 150 models were trained and analyzed.

### 3.5. Evaluation of the Discovered Knowledge

In this step, the performance of previously trained data mining/machine learning algorithms is evaluated and the best performing model is selected. There are two main approaches for model evaluation: by reusing the previously collected dataset, or based on a newly collected dataset. The first approach assumes that separate data can be obtained for testing, which is only feasible if the data collection process can easily be repeated [80]. In the second approach, the assumption is that only one dataset for both testing and training is available which should be split into a training and a testing set. Generating the two sets may be done by simply selecting a random split of the data (e.g., 70% for training and 30% for testing). In most practical situations, the dataset is separated into a number of equally sized chunks, so-called folds, of instances. The algorithm is then trained on all but one of the folds. The remaining fold is then used for testing. This process is repeated several times so that each fold gets an opportunity to be left out and act as the test set for performance evaluation. Finally, the performance measures of the model are averaged across all folds. The process is known as k-fold cross validation.

The performance evaluation of a regression model is different from that of a classification model. For evaluating the performance of a regression model, an error (or cost) function is typically used [123]. This function compares the actual values (targets) that are known with the values predicted by the algorithm and gives a measure of the prediction error (or the distance between the actual values and the predicted values). Often, this cost function is the same as the one used internally by the algorithm for optimising the model coefficients/parameters during the training phase. Examples of such functions that are commonly used are: Root Mean Squared Error, Relative Squared Error, Mean Absolute Error, Relative Absolute Error, Coefficient of Determination, *etc.* [80].

For evaluating the performance of a classification model the prediction error is commonly calculated based on the misclassification error metric, which gives a binary output by simply testing whether each prediction was correct or not (*i.e*., 1 or 0). This binary output is used to compute a confusion matrix that contains the true positives and true negatives (*i.e*., percentage of correctly predicted instances), false positives and false negatives (*i.e*., percentage of instances that were incorrectly labeled). Various performance metrics including precision/recall, F1 score, *etc*. (explained later in Section 4.6), are derived based on the confusion matrix.

Finally, the best performing model with respect to the considered performance metric is selected for both regression and classification—also referred to as model selection. When the performance of the models is not satisfactory, the knowledge discovery process returns to the previous two steps where typically better feature engineering and model tuning are performed.

For instance, in [9] one dataset was used for training and testing: 60% of the total dataset instances were randomly selected for training, while the remaining 40% for testing. The mean square error (MSE) was used to reflect the average misclassification error and evaluate the performance of the following algorithms: Bayes classifier, logistic regression and artificial neural networks (ANN). They tuned the feature space through feature engineering for: *RSSI*, *SNR*, *LQI*, *PRR*, and the system parameters by changing values for: *W*, *L*, *P* and *I* as discussed in Section 3.4. For each feature/parameter combination the authors trained and evaluated every algorithm again in order to select the best model. The best performing model turned out to be logistic regression trained on the feature vector ${\left[PRR,LQI\right]}^{T}$. To show the advantages of the model an evaluation against existing solutions such as 4Bit and STLE was also performed.

### 3.6. Using the Discovered Knowledge

A standalone offline machine learning system might not be a very useful tool. Therefore, in the last KD step, the software development process of the selected model is considered. As in any traditionally software development process, after the initial analysis that was performed through the previous steps, an appropriate design describing how the model should work is proposed, which may be visualized by a simple block diagram or more detailed with several UML diagrams. Typically, it is also considered how to integrate the proposed model with existing systems (e.g., into existing environment of the target platform, with existing database management system, with existing visualization tools, *etc*.). Then, a prototype program of the model is implemented on the target architecture, by means of coding using adequate programming language which may depend on the target platform. The implementation is verified and validated through several tests. At the end, an implementation of the model is deployed at the target system.

For instance, after the initial analysis and several experiments performed based on offline trained models, Liu *et al*. [9] proposed a design for an (online) implementation of their logistic regression trained model, called *4C*. Their target platform was a Tmote Sky mote running TinyOS [124]. To this end, the authors had to consider how to integrate their model with the existing environment in TinyOS (e.g., with the forwarding engine, routing engine, link estimator *etc*.). Then, they implemented the logistic regression-based link estimator as a module in nesC, which is the programming language in which the TinyOS system is written. At the end, they deployed the *4C* module online on wireless sensor motes and tested through several experiments against existing link quality estimator implementations like STLE and 4Bit and demonstrated superiority of their solution.

### 3.7. Examples of Using Data Science in Wireless Networks

In traditional wireless research, research often starts from theoretical models to design solutions which are then evaluated or validated using a simulator or experimental setup. For example, in [52], a spectrum usage prediction problem for cognitive radios is simulated in which neural networks are used as the machine learning algorithm to predict which unlicensed channels are idle. In contrast to these traditional research approaches, the research works below [9,11,12] take a truly data-driven approach, *i.e*., starting from large, real-life wireless datasets to extract knowledge about wireless systems. To this end, this section provides an overview of selected applications of data science approaches in wireless networks by clearly identifying each step of the knowledge discovery in their methodology and parts that do not follow best practices. We hope in this way to help the reader better understand how to properly apply each step of the KD framework to existing problems originating from wireless networks, and at the same time, motivate for new research ideas in the wireless domain.

#### ***Use Case 1:*** *Link Quality Estimation*

Liu *et al*. [9] improved multi-hop wireless routing by creating a better radio link quality estimator. They investigated whether machine learning algorithms (e.g., logistic regression, neural networks) can perform better than traditional, manually-constructed, pre-defined estimators such as STLE (Short-Term Link Estimator) [104] and 4 Bit (Four-Bit) [105]. The methodology employed in their work followed all the steps from the knowledge discovery process and we used examples from this work to show how the KD process steps can be successfully applied to wireless networks in previous parts of this section. They clearly formulated the wireless networking problem and the corresponding data mining/machine learning problem, then they collected a large amount of data, analyzed it and made sure it can solve the problem, pre-processed it and fed it to the machine learning algorithms. Finally, they evaluated the way the algorithms performed at predicting the quality of the link and implemented the most promising one (in this case logistic regression) on several testbeds. While in [9] the authors used an off-line trained model to do prediction, in their follow-up work [10], they went a step further and both the training and the prediction were performed online by the nodes themselves. The advantage of this approach is that the learning and, thus the model adapt to changes in the wireless channel, that could otherwise be captured only by re-training the model offline and updating the implementation on the node.

#### ***Use Case 2:*** *Traffic Classification*

Crotti *et al*. [11] proposed a new method for identifying the application layer protocol that has generated a particular traffic flow. Their approach was motivated by the fact that standard techniques at the time often failed to identify the application protocol (using transport layer ports) or scaled poorly in high-capacity networks (detailed analysis of the payload of each packet). Their method is based on the statistical properties of the traffic flows which allows discrimination between traffic classes. While they do not seem to define their problem explicitly as a data mining/machine learning problem, nor use well-known data mining/machine learning algorithms, they took a truly data-driven approach, they used methodologies and terminologies from data science and followed all the steps of the KD process except the last one, *i.e*., system implementation. The authors clearly identified and formulated the application protocol identification problem as a (traffic) classification problem, where the classes are the application protocols learned by the classifier (e.g., HTTP, SMTP, SSH, *etc*.). Then, they collected data for training from their faculty’s campus network and created the feature vectors (pairs of {*s*, Δt}, where *s* is the packet size, and Δt the inter-arrival time between successive packets) and the training set, pre-processed it using a Gaussian filter to reduce the effects of noise and fed it into their custom learning algorithm for training. The trained model consists of pairs of a matrix, $M_{p}$ and a vector $V_{p}$, for each protocol *p*, where $M_{p}$ is the *protocol mask* that captures the main statistical properties of training flows produced by the same protocol, while $V_{p}$ the protocol specific threshold that is used by the classifier to determine how close an unknown flow is to a fingerprinted protocol with mask $M_{p}$. At each step they explained the rationale behind each action taken, which may be a replacement for the data analysis phase. At the end they evaluated their model with a separate test set collected from the same network.

#### ***Use Case 3:*** *Device Fingerprinting*

In [12], the authors proposed a new solution for fingerprinting wireless devices that may help existing *network access control* (NAS) systems enhance network security by allowing access only for certain devices or device types in accordance with a defined security policy. Unlike traditional security mechanisms that rely on device authentication based on public key cryptography and digital certificates, that can be simply transferred to another device, their approach relies on differentiating devices by looking into the statistical distribution of inter-arrival times between packets generated by the same device and a particular application. Their assumption is that the physical particularities of devices such as processor, DMA controller, memory, clock skews, *etc*. reflect on how these devices send packets over the air. By monitoring the inter-arrival times, it should be possible to distinguish individual devices or devices of the same type (*i.e*., devices that have the same hardware configuration), thus they formulated a classification problem. They collected the necessary data to prove their hypothesis from two testbeds: an isolated network environment and a live campus network. Their feature vectors consist of histograms constructed from inter-arrival times and their label or target values consist of discrete classes (e.g., Dell Notebook). They selected an artificial neural network (ANN) model that was trained offline and then evaluated on the testing data. The best performing model turned out to be an ANN model with one hidden layer of 50 nodes. Even though this research also followed all the KD steps, some of the individual steps do not seem to conform with existing best practices. For instance, it is not clear how the feature vectors were generated in the data pre-processing step. It is also unclear how many training examples were used in the data mining part and how the training and testing sets were separated. Finally, the evaluation is presented in a biased manner by only showing an example of a well-performing case using only a subset of the relevant measures. In the following Case study section, we will use this publicly available dataset to provide an in-depth illustration of how the steps of the KD process should be performed.

Tumuluru *et al*. [52], as earlier mentioned, define a spectrum usage prediction problem. The authors follow some of the steps of the KD process. They used neural networks as the machine learning/data mining algorithm, but they only use synthetic data produced with a simulator and no real-world data. This means that the data understanding step is not relevant in their case since they know a-priori the statistical details of the synthetic data. This is in contradiction with the discussion in Section 3 regarding the KD process. Additionally, their evaluation is far from the best practices used in the machine learning community. To remedy such cases, the next section aims to further close the gap between wireless researchers and data science experts by giving very concrete examples and considerations for each of the knowledge discovery steps.

## 4. Case Study

This section details the implementation of each component of the general-purpose framework introduced in Section 3. These implementations are used to demonstrated how a real-life wireless networking problem can be solved using a data-driven approach. However, note that the presented techniques are generic, as such are not limited to the particular problem use case. The same generic algorithms and techniques that are introduced in each step as well as the overall methodology can be applied to a different type of problem. The main difference relies in the data mining step, where the practitioner has to select specific algorithms that are suitable to solve the previously identified type of problem. Recall that Section 2 guides the reader how to properly formulate a specific wireless domain problem into a data science problem with a list of suitable algorithms.

### 4.1. Solving a Classification Problem in Wireless Networks

For the case study, we chose a simple *classification* problem about *wireless device fingerprinting* which was introduced by Radhakrishnan *et .al*. [12] for which all the data (*i.e*., the *GaTech* dataset) is publicly available on the CRAWDAD repository [24]. The dataset was also produced as part of the work presented in [12]. It can be downloaded by anyone who wishes to replicate the KD process steps further detailed in the remainder of this tutorial. However, while the GaTech dataset was used in [12] to solve the wireless device fingerprinting problem using a custom approach, this tutorial paper reuses the same dataset to demonstrate the correct methodology of applying data science techniques for solving a wireless domain problem through a practical approach. Finally, a brief comparative study of the proposed methodology compared to their custom approach highlights the importance of applying the identified best practices developed by the data science community.

The scripts used during this tutorial for implementing the individual components of the framework can be downloaded from [23] together with all intermediary and processed datasets, thereby offering interested readers the opportunity to quickly learn how to apply these or similar steps themselves.

#### Wireless Device Fingerprinting: A Brief Overview

Due to the broadcast nature of the wireless transmission medium, wireless networks are vulnerable to various network security attacks; in particular, they are easy to compromise in terms of their service availability (through Denial-of-Service attacks) as well as data confidentiality, data integrity and user/device authentication (wireless security protocols are known to have security holes that can be easily exploited). The major security thread facing wireless networks is node forgery, where an attacker obtains the credentials (cryptographic keys) of the legitimate user and impersonates the trusted node. One promising approach to reduce the vulnerability of wireless networks to these attacks is device fingerprinting [125]. Device fingerprinting is the process of gathering device information (passively or actively) to extract unique device-specific signatures or fingerprints that can be used to reliably and robustly identify devices. A device identification system typically examines a three-step process that consists of (i) identifying the relevant patterns; (ii) extracting patterns from raw data; (iii) creating a model for device identification. The extracted patterns from the raw observations are also called features. Different approaches utilize different device-specific features, which can be categorized based on the layer of the protocol stack from which the information has been obtained.

1. *PHY layer features:* PHY features are derived from the RF waveform of the received signal. The most common PHY layer information for device fingerprinting are RSSI measures. However, the RSSI depends on the transmission power, propagation of the signal and attenuation imposed by the channel. More fine-grained features are channel state information at the receiver (CSIR) [126,127], channel frequency response (CFR) [128,129], channel impulse response (CIR) [126,130], carrier-frequency difference (CFD) [131], phase shift difference (PSD) [131], second-order cyclostationary feature (SOCF) [131], I/Q signal samples [132], *etc*.
2. *MAC layer features:* The motivation behind utilizing MAC layer features for device identification is that some of the MAC layer implementation details are not specified in the standard and are left to the vendors. Therefore, MAC layer features are usually vendor specific. Some example works are: observing unique traffic patterns on the MAC layer to detect unauthorized users [133,134], observing the clock skew of an IEEE 802.11 access point from the Time Synchronization function (TSF) timestamps sent in the beacon/probe frames [135]; MAC features such as transmission rate, frame size, medium access time (e.g., backoff), transmission time and frame inter-arrival time [136].
3. *Network and upper layer features:* Features at the network and upper layers typically look into user’s traffic patterns or inter-arrival times calculated at the network and application layer. For instance, in [137] Gao *et al*. and in [12] Radhakrishnan *et al*. use inter-arrival times from TCP and UDP packets as features. Ahmed *et al*. [138] uses traffic patterns of digital TV broadcasting to identify devices. On the other hand, in [139] Eckersley uses higher layer features by tracking the web browser behaviour by analyzing the browser’s requests/replies.

Finally, multiple features can be combined to form a device fingerprint, leading to a cross-layer identification approach. The case study example of this paper falls into the last category, where inter-arrival times based features are used as is explained in next section.

### 4.2. Understanding the Problem Domain

One of the challenges of the knowledge discovery process is that the data scientists having the skill set and experience necessary for carrying out the process are not necessarily experts in the areas for which they apply these techniques. For instance, they will know what terminology such as supervised and unsupervised methods, 10-fold cross-validation, *etc*. means and what techniques should be used to execute them, but they will not know what packet inter-arrival time, TCP, UDP and SCP traffic are in detail. On the other hand, it requires quite some time and effort from a wireless networking expert who is familiar with terminology such as SCP traffic, to carry out the knowledge discovery process because they will have to dive into and learn all the new data science terminology. This learning path also has many alleys and, for a novice, many aspects could turn out to be confusing.

As discussed in Section 3.7, there are several research works which seem to take a data science approach in wireless networks while they miss some fundamental steps or parts of the KD process.

#### 4.2.1. Understanding and Formulating the Device Fingerprinting Problem

The device fingerprinting problem defined in [12] is an actual problem related to the real-time detection of malicious devices in wireless networks that aims to classify wireless devices based on several types of traces. It is based on the argumentation that each device exhibits some traits inherent with its physical implementation. As a result, it is assumed that the packet creation process varies across different device architectures and is influenced by the CPU, L1/L2 cache, DMA controller, physical memory, *etc*. At the same time, similar variations exist between individual devices with the same architecture, due to differences of clock skews. Thus, it might be feasible to differentiate individual devices and also cohorts of devices with the same hardware characteristics (*i.e*., device types).

Furthermore, it should be possible to determine and learn the differences between the devices and their types by looking at packet inter-arrival times (IAT). This implies that, at capture time, for each packet it is known which device sent it (from IP address) and what transport and application layer protocol sent it. To verify this hypothesis, the capture of IP traffic flows from several target devices is required. In the case of active device fingerprinting, where devices are queried, e.g., with ICMP echo request packets (Ping), the captured packets may contain information about the MAC address of the target device. However, it should be clear that the MAC address contains only the unique identifier of the manufacturer of the device, which is not sufficient to identify a particular device nor its type. On the other hand, MAC addresses can be easily spoofed.

Based on this argumentation, the following hypothesis can be constructed: "by building per device and per device type models based on packet IATs, it should be possible to *classify* devices and device types as well as their normal behaviour". Thus, the problem of fingerprinting wireless devices can be translated into a data mining classification problem. To verify the stated hypothesis data has to be collected to find out:

- How much can data from one device (e.g., a Dell Netbook) tell about the data from other similar devices (e.g., other Dell Notebooks)?
- How much can a certain type of device (e.g., Dell Netbooks) tell about other device types (e.g., iPhones)?

By answering such questions using the collected data, we should be able to anticipate whether the data can validate our hypothesis and further process with the KD process. If it turns out that the data cannot be used to validate the hypothesis, we have to go into an iterative process that involves (1) tuning the hypothesis and (2) collecting different data.

#### 4.2.2. Collecting the Data for Validating the Hypothesis

In order to validate their hypothesis, Radhakrishnan *et al.* [12] used three traces with packet inter-arrival times collected from several devices and several protocols in two different testbeds: an isolated testbed and a real testbed. For the purpose of this tutorial, we focus on the isolated testbed data that contains 94 files containing IATs from 14 different devices and four different combinations of tools and protocols as summarised in Table 3. Four different device types (Dell Netbook and iPad; iPhone and Nokia phone) were used in the testbed containing five pieces of Dell Netbook, three pieces of iPad, four pieces of iPhone and two pieces of Nokia Phone. The collected data consists of TCP, UDP and ICMP traffic generated using three tools: iPerf, Ping and SCP. The detailed device specification and other detailed information about the experimental settings under which the data has been collected are available in Tables 1–3 in [12].

Table 3 presents a summary of IAT traces that were collected for each combination of device, protocol and tool. For instance, in the first line of the table it can be seen that five Dell Netbooks were used in the experiments as follows. First, five TCP traffic traces were generated using the iPerf tool. These five traces had a minimum of 841.299 instances (*i.e*., IAT values) and a maximum of over 3 million such instances. The average number of values in the dataset is 1.8 million with a standard deviation of 948.900. Second, the UDP traffic was also generated using iPerf, but in this case, three different packet size/rate settings were used (Case 1: 64 byte packets at 1 Mbps, Case 2: 1400 byte packet at 1Mbps and Case 3: 64 byte packets at 8Mbps according to Table 1 in [12].), which is why the table mentions three cases, and five traces for each case, totalling 15 traces with a minimum of 298.956 items and a maximum of 5.702.776 items per file. Also Ping ICMP traces were collected, albeit only for three of the five Dell Netbooks, and SCP TCP data as shown in the corresponding lines of the table.

It can be seen that the number of traces is consistent with the number of devices for each round of the data collection. Except for the Dell Netbook, the same number of devices have been used throughout all the experiments. For the Dell Netbook, the Ping ICMP collection only used three devices, while all the remaining campaigns used five. Furthermore, for some experiments, traces for several cases have been collected using various packet size and rates. Overall, the data is stored in 94 files containing a total of 137,348,241 data points, with an average of 1,461,161 data points per file, a median of 1,311,227 and a standard deviation of 1,315,710. The standard deviation is relatively high as some files contain several million data points (*i.e*., iPhone iPerf UDP case 3, trace 3 contains 4,057,490 data points) while some other files contain several hundred thousands (*i.e*., Dell Netbook iPerf UDP case 1, trace 1 contains 299,521 files). We can anticipate that having unbalanced traces in terms of the amount of data may lead to smaller training/testing sets for some devices and larger for others. In some cases, this may mean that the models where more training/testing data is available might capture the underlying process better than others [140].

##### Practical Considerations for Understanding the Problem Domain

Once the wireless problem and its potential data-driven solution has been identified, a relevant body of data has to be collected, processed, and understood to validate the stated hypothesis.

To facilitate the data collection process, readers can utilize repositories with existing datasets, or collect new traces from publicly accessible wireless testbeds.

*Repositories* provide access to data from real-life wireless experiments. Examples are CRAWDAD [141], CONAN project [142], the "UC San Diego wireless measurements" whose dataset is available at [143], the University of Washington "Wireless Measurements Project" [144], EVARILOS [145], "UMass Trace Repository" [146], *etc*.

*Wireless testbeds* allow to setup a specific wireless scenario and collect the relevant data to evaluate a hypothesis. Several testbeds are part of the **FIRE** federations [147] including: w-iLab.t [148], TWIST [149], IRIS [150], LOG-A-TEC [151], NITOS [152], *etc*., and **GENI** federations [153] such as: Motelab [154], ORBIT [155], Kansei [156], NetEye [157], *etc*.

### 4.3. Understanding the Data

As briefly discussed in Section 3.2, this second step of the KD process validates the data and the hypothesis. It verifies whether the data has the potential to lead to an accurate and meaningful model. It also verifies whether it makes sense to proceed further with the initial problem definition, or if there is a need for a reformulation. Typically, this step also identifies whether data has to be pre-processed. This step is sometimes referred to as Exploratory Data Analysis (EDA) and it consists of applying analytical/computational and graphical/visualization techniques on the data [158].

#### 4.3.1. Generic EDA Techniques

Most EDA techniques rely on statistical methods, tools and techniques. For this reason, EDA is also known as the “statistics” part of the KD process. In contrast to the classical statistical hypothesis testing that verifies certain a priori assumptions (e.g., correlations between different attributes/variables, where there is some information concerning a possible dependency), EDA uses more sophisticated techniques to identify systematic relations between attributes/variables about which no prior information exists. Data may be analyzed using computational or visual techniques.

- Computational techniques utilize statistical distributions, five-number summary, coefficient of determination, advanced multivariate exploratory techniques (e.g., cluster analysis, principal components and classification analysis, classification trees, self-organizing maps, *etc*.). In this tutorial, we use the five-number summary and the coefficient of determination to guide the reader through the process of understanding the data. More advanced techniques can be adopted from the domain specific literature [159].
  The five-number summary consists of five reference values that summarize the behavior of a dataset: min—the minimum value, $Q_{1}$—first or lower quartile (the middle number between the minimum value and the median), median—the “middle number” of the sorted dataset, $Q_{3}$—third or upper quartile (the middle number between the median and the maximum value), and max—the maximum value.
  The coefficient of determination (denoted by $R^{2}$) is a simple statistic frequently used for determining relationships between system variables. It is defined as:
  (2) $$R^{2}=1-\frac{\sum_{i}{\left(y_{i}-f_{i}\right)}^{2}}{\sum_{i}{\left(y_{i}-\bar{y}\right)}^{2}}$$
  where $y_{i}$ is the target value, $f_{i}$ is the value modeled (predicted) by a linear function *f*, while the denominator represents the total variation of the target variable’s instances.
  In general, $R^{2}$ describes how well some of the data can be approximated by a regression line constructed from some other data (*i.e*., one feature from the feature vector). High values of $R^{2}$ scores will indicate that there is a high linear dependency between a particular feature and the target value, while low values of $R^{2}$ may indicate the opposite.
- Visual techniques utilize histograms, box plots, scatter plots, contour plots (for functions of two variables), matrix plots, *etc* [160]. Histograms, also used throughout the rest of the tutorial, reflect the frequency density of events over discrete time intervals. They help understand and graphically verify obtained results [161]. For instance, they display the distribution, the mean, skewness and range of the data. They are also a useful tool for identifying deviating points which should perhaps be removed from the dataset. A practical feature of histograms is their ability to readily summarize and display large datasets.

A typical scenario for data exploration starts with computing simple statistics like the five-number summary and getting more insight in the data using simple visualisation tools such as histograms. These are simple but powerful techniques that help understand different data properties, find patterns in data, explore if there are instances out of compliance and suggest further modeling strategies.

#### 4.3.2. Applying EDA Techniques to the GaTech Data

##### Validating the Fingerprinting Data

To exemplify this step of the KD process, we focus on the iPerf TCP traces from all devices and device types, but of course the same procedure may be applied to the remaining traces. Exhaustively considering all traces would lead to a very long paper with no significant added value. We observe 14 iPerf TCP traces from: 5 Dell, 3 iPad, 4 iPhone (3G and 4G) and 2 Nokia devices. Table 4 presents the five-number summary for TCP IAT data. It can be noticed that the dataset from the iPad1 and iPad2 have significantly higher maximum values compared to the others. The iPad2 has a maximum IAT value (10.36 s) that is 100 times higher than the maximum IAT value of iPad3 (0.11 s). iPad1 has the next highest value of 5.2795 s, followed by iPhone3G1 with an IAT value of 2.95 s. The values for min, Q1, median and Q3 are more balanced, suggesting that only a few high-valued data points are deviating from the rest of the dataset. Furthermore, the Q1, median and Q3 values are lying close to each other for each dataset, while the max values are distant from Q3, suggesting that the majority of the measurements and the relevant information lies in the IAT measurements with values lower than Q3. Consequently, all the points strongly deviating from Q3, *i.e*., close to the max points, might be potential outliers. Those distant data points deviate from a normal IAT trace pattern for a particular device, and they may have occurred due to measurement errors.

**Figure 2:**
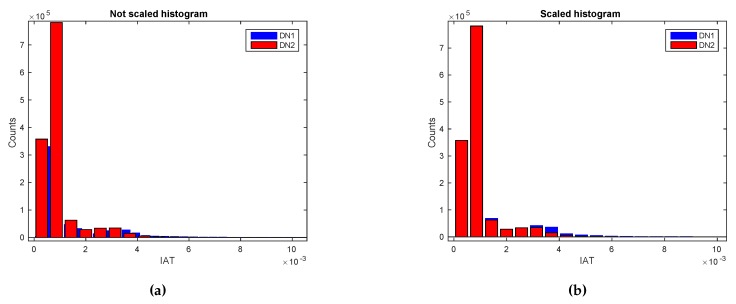
Effect of non-scaled histograms for DN1 and DN2 with 500 bins. Due to their different minimum and maximum values, the bins of the histograms from DN1 and DN2 do not use the same interval values. (**a**) Non-scaled histograms; (**b**) Scaled histograms.

Table 4 reveals large variations of min and max values between the datasets. In other words, the datasets span different ranges, which will result in histograms with nonconforming bin widths. Figure 2a illustrates this situations for histograms created from two datasets (DN1 and DN2) with a fixed number of bins. Such histograms have to be adjusted in order to perform a correct prediction task in the following steps. This can be done by adjusting the start and the ending points of the histograms, so that the bins for all histograms have the same width and can be compared against each other. This has to be done for all the 14 datasets from Table 4. We implemented a simple algorithm that looks at the max point over all histograms, which according to Table 4 belongs to device iPad2, and aligns all histograms with regard to that point, by padding the dataset with a new zero-count max value as a place-holder. Figure 2b illustrates the same two histograms from Figure 2a after aligning.

After adjusting the histograms, the required histogram granularity has to be identified. For this, typically a trade-off between accuracy and computational efficiency has to be made. A very high number of bins will approximate the distribution more accurately, however, as a consequence the computational burden of a prediction algorithm that has to process a high-dimensional input might increase significantly. On the other hand, due to the difference in range span between datasets from different devices, after adjusting all histograms to the same max point, it turns out that datasets that had a much lower max point compared to the new aligning max point will contain only a few number of (non-zero) bins for a low granularity (e.g., 500 bins). For instance, the original endpoint of the histogram of device DN1 (0.1872 s) will be shifted to the maximum endpoint of all dataset which corresponds to iPad2 (10.36 s). After creating a histogram for DN1 with granularity of e.g., 500 bins, the resulting distribution will contain only 10 non-zero bins, each having a width of 0.02 s. This is due to the fact that most of the IAT values of DN1 appear in the range of the first bin, *i.e*., [0-0.02], therefore almost all data points fall within this single bin. Similar remarks can be made for histograms belonging to other devices. Such one-bin histograms insufficiently estimate the real distribution of the data by introducing high discretization error. Discretization error can be reduced by increasing the number of histogram bins. Therefore, we analyzed also histograms with granularity of N=5000 and N=50,000 bins. The resulting histograms will have a smaller bin width of 0.002 s and 0.0002 s respectively, hence finer granularity and smaller discretization error. A further increase of histogram granularity would lead to even better probability density estimates, but at a higher computational cost. On the other hand, higher granularity introduces more variations and differences between histogram, even between histograms from the same device type. Hence, for the data analysis we choose *N* = 50,000, which gave a sufficiently good approximation of the underlying data distribution.

However, it should be noted that for a classification system that makes predictions based on histogram instances, this might not be a suitable number because of training time and model complexity issues. Section 4.4 discusses how to deal with imbalanced data, detect and remove outlying points and overcome expensive training models. In order to make a clean separation between the data analysis and the data pre-processing step, we leave the data as is, without removing the deviating points (later in Section 4.4 introduced as data cleaning).

##### Validating the Fingerprinting Hypothesis

In order to validate their hypothesis based on the collected data, Radhakrishnan *et al*. [12] performed an analysis by using simple visual techniques in which they show that: (1) the probability distribution functions (PDF) that approximate the distribution of the empirically collected data vary for devices with different internal hardware characteristics, which may enable device type classification; (2) the average IAT values vary between devices stemming from the same device type (e.g., Dell netbooks with the same hardware configuration) because of clock skew, which should enable individual device classification. As a result of the above made observations, the data is considered suitable for the hypothesis and it seems that the distribution of the data rather than its shape (*i.e*., trend, periodicity, *etc*.) are relevant for the problem. An analysis of whether useful information is also contained in the shape of the data is missing in the original paper, possibly because it is not relevant for their study. However, generally speaking, analyzing such information may be useful for improving the overall performance of the classifier [162].

In addition to visual techniques, used in [12], computational techniques are also very relevant to answer questions about the characteristics and relationships between the underlying data. In particular, when the aim is to find the predictive power of one trace with respect to others.

(i) How much can data from one Dell Notebook tell about the data from other Dell Notebooks?

Table 3 has shown that there is quite some data about Dell Netbooks (DNs), therefore, we will first try to understand how many IATs captured from one Dell Netbook can be used to predict the behavior of other such netbooks. Table 5 presents the $R^{2}$ coefficient for the iPerf TCP IATs with highlighted traces from the five Dell Netbooks.

**Figure 3:**
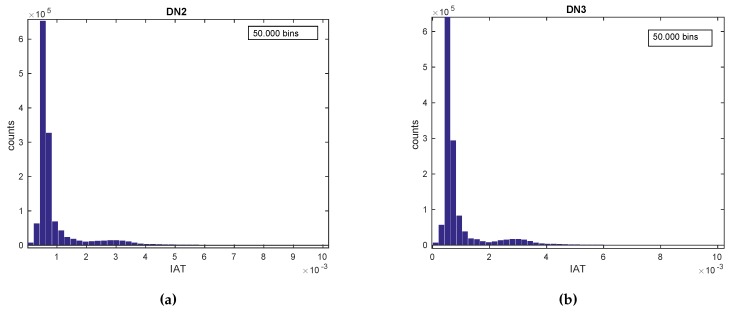
Comparing histograms for DN2 and DN3 for TCP case. (**a**) Histogram of DN2; (**b**) Histogram of DN3.

It can be seen that the traces from DN2 and DN3 have a very high determination coefficient of 0.998, meaning that their distributions are very much alike. To illustrate the similarity of these traces, Figure 3a,b present the scale-adjusted histograms for DN2 and DN3. For a regression task, this is good, meaning that data from one of the two devices can be used to predict data from the second device. For a classification task, this may mean that data coming from these two devices are more likely to be considered as belonging to the same class. In this case, it means that classification methods might have trouble differentiating between DN2 and DN3.

A high determination coefficient can also be found between data from DN4 and DN5 with an $R^{2}$ value of 0.958. There is also a high determination between DN2 and DN4 and DN3 and DN4, followed by DN2 and DN5 and DN3 and DN5, thus similar conclusions can be drawn for these.

**Figure 4:**
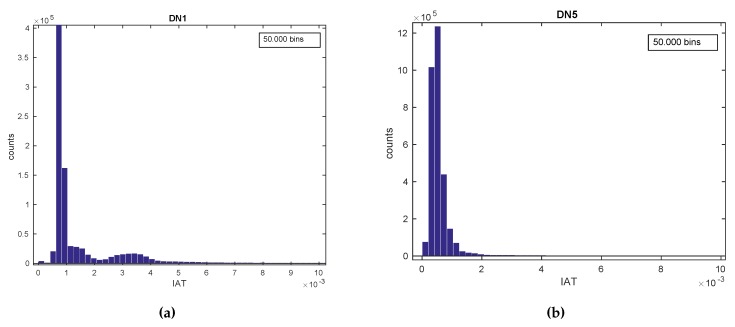
Comparing histograms for DN1 and DN5 for TCP case. (**a**) Histogram of DN1; (**b**) Histogram of DN5.

Table 5 also shows that the determination coefficient considered for DN device pairs that include DN1 have a very low $R^{2}$ value (first table row or column) with the lowest $R^{2}$ value of 0.0967 for the (DN1, DN5) pair and the and highest for the (DN1, DN2) pair with an $R^{2}$ value of only 0.241. Figure 4a,b present histograms for DN1 and DN5 which had the lowest $R^{2}$ score. It is clear that the histograms differ much more than in the previous case, especially in the tails part, the shape and the peak values.

In conclusion, we expect that data coming from device DN1 will not be confused with data from other devices for the classification task, however a regression task would perform poorly at predicting the data generated by this particular device. $R^{2}$ results for the other traffic cases can be obtained from this repository [163].

ii) How much can iPerf TCP data from a set of similar devices tell about the same data from other devices?

Next, instead of looking at individual devices, we look at TCP traces containing aggregated data from multiple device types. Table 6 presents the $R^{2}$ scores for data collected for sets of similar devices (*i.e*., from same device type) for the case of TCP traffic. This information can be used to determine whether TCP data can be used to identify the type or device that transmitted the data. Dell Netbooks and Nokias have an $R^{2}$ value of 0.8235, meaning that a regression task would perform well predicting IAT values for Nokia based on training values for Dells, however a classification task might be confusing the two classes, unless it can find a way to discriminate based on the training data. Similar conclusions are true for iPad and Nokia devices which have an $R^{2}$ value of 0.7359, followed by iPad and iPhone with $R^{2}=0.6562$, and Dell and iPad with $R^{2}=0.6254$. $R^{2}$ results for the remaining traffic cases can be obtained from this repository [163].

##### Practical Considerations for Understanding the Data

To understand the data gathered in previous step, the practitioner can make use of several existing data mining toolboxes/libraries depending on the programming language of preference including R [164], MATLAB [165], Octave, Weka [166], RapidMiner [167], Rattle GUI [168], and several Python libraries such as Matplotlib, IPython, Pandas, NumPy/Scipy, scikit-learn, *etc*.

### 4.4. Data Pre-Processing

The goal of this step is to transform the collected data, also referred to as raw data, into a format suitable for feeding into data mining and machine learning algorithms, also referred to as training data. Section 3.3 introduced four pre-processing sub-steps that can be used to prepare data for training. This section clarifies how each sub-step should be performed, so as to improve the efficiency of the mining process and quality of the mining results. One of the most important aspects during this process is indisputably feature engineering. Feature engineering aims to find a representation of the data, using domain knowledge, that is most likely to solve the problem defined in the first step of the KD process. In this section of the tutorial, we exemplify the data cleaning, data transformation and data reduction methods to guide the reader through the process of pre-processing and feature engineering.

#### 4.4.1. Generic Data Pre-Processing Techniques

**Figure 5:**
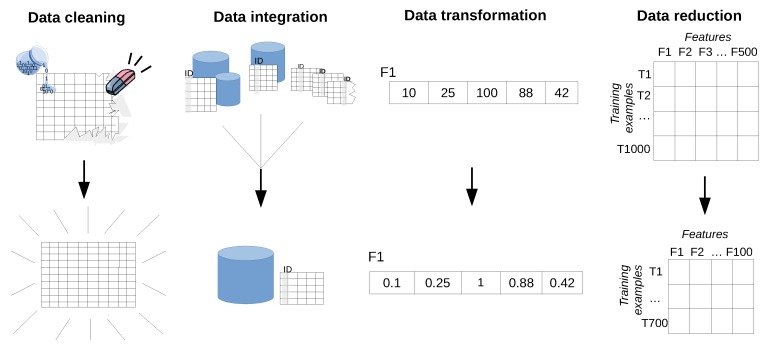
Data Pre-Processing Steps.

Figure 5 illustrates how each sub-step of data pre-processing should be performed.

**Data cleaning**, as illustrated on Figure 5, fixes data by detecting/removing outliers, filling in missing values or resolving inconsistencies [169]. Outliers may be detected using simple visualization techniques like boxplots and histograms, by simply ordering data and declaring all values above or below a user-defined threshold as outliers (requires domain knowledge), or some more sophisticated techniques such as clustering where similar values are organized into clusters, while the ones falling outside of the clusters may be considered as outliers. Missing values may be filled for numerical values by interpolation, while for nominal values by majority voting. Other options may be: fill the missing value for a given feature by the mean (or median) of all known values of that feature, or use a learning algorithm (e.g., decision trees, *k*-NN, *etc*.) to predict the missing value [170]. One of the most common and successful techniques for imputing missing values is to use the *k* nearest neighbors (*k*-NN) algorithm, where for each missing attribute the goal is to find the k nearest neighbor samples having that attribute. Inconsistencies may be solved with the help of domain knowledge and deeper analysis of the problem. In this tutorial, we will perform data cleaning by removing outliers above a pre-determined threshold.

**Data integration**, as illustrated on Figure 5, combines data from several disparate sources. These sources usually include datasets stored in multiple databases. Typically, such datasets are merged by matching based on some ID or database attribute. After merging, the data may be reduced in order to remove redundant information. For instance, one attribute may be redundant if it can be somehow derived from or replaced by another attribute or set of attributes. Redundancy may be detected by measuring how strongly one attribute implies the other, with some simple statistics like the correlation coefficient for numerical data, and the $\chi^{2}$ (chi-square) test [171] for nominal data. In this tutorial, data integration is not further considered, because it is not applicable to the target problem and in general has found less use for problems concerning wireless networks.

**Data transformation**, as illustrated on Figure 5, is typically performed by either *min-max normalization* or *zero-mean normalization* (also known as *standardization*). *Min-max normalization* scales the features down to the unit range, *i.e*., [0,1], by the following transformation: $\frac{\left(x_{i}-Min\right)}{Max-Min}$, where $x_{i}$ denotes the feature value, while Min and Max the minimum and maximum value of the feature, and Max-Min the range of the feature. *Zero-mean normalization* scales the features from original scale to standard deviation scaling, by the following transformation: $\frac{\left(x_{i}-\mu_{i}\right)}{\sigma }$, where $\mu_{i}$ denotes the feature mean and *σ* the standard deviation of the feature. This transformation scales the features to have zero mean and unit standard deviation.

Transformed data can help improve the accuracy and the efficiency of the data mining algorithms. For instance, distance based algorithms such as *k*-NN (see Section 4.5) provide better results on normalized data, because distance measurements taken on features with larger values are likely to outweigh distance measurements taken on features with smaller values, which may worsen the accuracy of the mining results [169]. For parametric algorithms, such as neural networks (see Section 4.5), it may help speed up the converging process in the training phase [172]. In this tutorial, we will use min-max normalization for neural networks and *k*-NN.

**Data reduction**, as illustrated in Figure 5, can be done by replacing the original data with a smaller subset of data, also known as dimensionality reduction, or by replacing data by smaller data representations, also known as numerosity reduction [169].

Dimensionality reduction typically refers to the process of selecting a subset of the original data features by removing irrelevant, weakly relevant or redundant features. In machine learning and data mining, this process is also known as feature selection [173]. Feature selection is considered an art in the field and there are several ways of performing it, from manual to highly automated by using simple of complex algorithms [174]. For instance, decision trees (see Section 4.5) can be used for selecting only the most relevant (informative) features. The algorithm forms a tree from the given data by selecting the most informative feature for partitioning the data at each node. The features that do not appear in the tree are assumed to be irrelevant and removed from the final feature set. For time-series data, dimensionality reduction can be achieved by applying the discrete wavelet transform (DWT) or the fast Fourier transform (FFT), which summarize a series of data points by extracting Wavelet or Fourier coefficients that describe the time-series signal. Furthermore, the data can be further reduces by also discarding features with high similarity as measured by the correlation coefficient [175].

Numerosity reduction is a different data reduction method that transforms the dataset into a compact representation (a model) of its main features. For instance, a parametric model may represented by a regression model used to estimate the data, so that only the model parameters (slope and y-intercept) need to be stored [176]. An example of a non-parametric model are histograms [177] which are used to reduce the representation of features, by storing only their approximate distribution (frequency count and bin width), rather than all individual data point values. In this tutorial, we will use histograms to represent the data, and form the training and testing sets for the data mining problem.

#### 4.4.2. Pre-Processing the GaTech Dataset

Section 4.3 revealed some interesting characteristics of the dataset. In particular, Table 4 showed that the datasets from several devices contain data points with values that are much larger than the rest of the samples. For instance, iPad2 has a maximum IAT value of 10.36 s, while its Q3 is approximately $10^{4}$ times below the max. Because such data points considerably deviate from the rest of the samples, they may be as outliers. Another problem encountered in step 2 was related to model complexity. Namely, because of imbalanced datasets, high granularity histograms with N=50.000 where used to still be able to capture the distribution behavior. However, histograms with granularity 50.000 would require a very complex and inefficient training process and final prediction systems. In order to solve both problems, existing outliers and model complexity, we use data cleaning techniques. The data analysis in Section 4.3 indicated that the main information that is necessary to correctly recognize a device is contained in the histogram body, while negligible or less relevant information for solving the target problem is contained in the tail. Hence, removing some of the highest-value data points should not negatively affect further mining results. Therefore, we perform data cleaning by first arranging the dataset values in ascending order, and then discarding values above a certain threshold (Th) to remove outliers.

In order to determine the optimal threshold, we perform the following test: we select several candidates for threshold (Th=0.3, Th=0.1 and Th=0.01) and evaluate the performance of the classifier trained on the resulting outlier-free data. We select the *k-NN* algorithm for the classifier trained for device type classification, cross-validation for performance evaluation and misclassification error as a measure to evaluate the classifier’s performance (see Section 4.6). Other options for determining the optimal threshold may be to test the closeness of the resulting distribution of the cleaned data to the distribution of the original data by means of statistical similarity measures such as coefficient of correlation and covariance, or perform a Kolmogorov-Smirnov test.

Removing outlying data points allows to train simpler models, using histograms with lower granularity. For instance, for Th=0.1 new histograms with much lower granularity could be created, without loosing consistency with the data analysis. If selecting 500 bins, the resulting histogram bin width will remain the same as it was in the data analysis for 50,000, *i.e*., $\frac{0.1}{500}=0.0002$. For this reason, we selected first the value Th=0.1 and use histograms with granularity 500. Using a smaller threshold will lead to histograms closer to the real underlying distribution at the cost of more data loss, while a higher threshold will lead to histograms that loose important aspects of the real distribution for the benefit of preserving more data points. To evaluate this trade-off we choose also a higher threshold value Th=0.3 and a lower Th=0.01. Table 7 depicts results in terms of achieved performance for the k-NN classifier and the corresponding average data loss (calculated on cleaned data over all devices) for data cleaning with thresholds Th=0.3, Th=0.1 and Th=0.01. It can be seen that selecting a threshold Th=0.3 leaves most datasets unchanged, *i.e*., the average data loss is 0.0032%. Changes occur only in datasets with high deviating points. By increasing the threshold more datasets become affected, and more data points discarded. For instance, when using Th=0.01 compared to Th=0.1, approximately 10 times more data is lost. However, the misclassification error tends to increase with the Th value, *i.e*., a smaller threshold may help improve the performance but at the cost of more data loss. The results can be explained by the fact that typically most information about a distribution lies in the body of the distribution, rather than in the tail. As such, the histograms for lower thresholds tend to better reflect the original distribution of the data and hence lead to better trained models.

We select the value Th=0.1 as a trade-off between data loss and performance gain. As an added benefit, this makes the forthcoming analysis consistent with the KD step 2 (Section 4.3). Since removing data above the threshold Th=0.1 and using 500 bins leads to histograms with same bin widths as in step 2, the overall distribution until the point IAT=0.1s from step 2 remains unaffected.

Table 8 shows the new $R^{2}$ results for the TCP traffic case only for Dell devices with cleaned data using Th=0.1. The table presents only $R^{2}$ for Dell devices because only those showed significant changes, with increased $R^{2}$, as can be anticipated when comparing to Table 5. This means that after data cleaning Dell Netbook devices show more similarities between each others histograms. $R^{2}$ results for other devices, as well as the results for device type analysis had minor changes; therefore, we omit their full presentation, especially since they are consistent with the discussion in Section 4.3.2. All results are available at [178].

**Figure 6:**
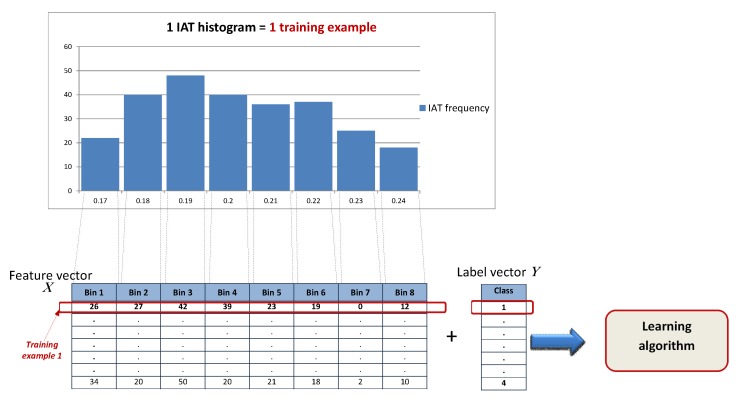
Explanation for training data design.

After data cleaning, data has to be prepared for training by defining the feature space and applying data transformation on the features. Figure 6 explains the feature design process. Histograms are turned into training instances, while the histogram bins into features. Note, representing data as histograms in this stage is a form of data reduction, as was explained previously in Section 4.4.

We define the number of IAT values falling within a particular histogram bin, e.g., frequency count (*fc*), as one feature. Thus, the first feature is the frequency count of the first bin ($fc_{bin_{1}}$), the second feature is the frequency count of the second bin ($fc_{bin_{2}}$), *etc*. In this way, we form the ***feature vectors***, defined as:

(3) $$X_{IAT}={\left[\begin{array}{c} fc_{bin_{1}},fc_{bin_{2}},\cdots ,fc_{bin_{N}} \end{array}\right]}^{T}$$

Figure 6 also shows that the training set consists of pairs ($x^{\left(i\right)},y^{\left(i\right)}$), where $x^{\left(i\right)}$ is a set of bins (features) from the same histogram (a feature vector), with $y^{\left(i\right)}$ the label for the associated class membership. As described, we use 500 bins, thus N=500 features. We use the Weka [166] data mining toolbox for training. The label vector, *Y*, is formed so that it takes values from the nominal set of device types {dell,ipad,iphone,nokia} and from the nominal set of devices {DN1,DN2,...iPad1,iPad2,...}, respectively. These labels can be used for training supervised learning algorithms (see Section 2.1.2).

After defining the feature vectors, we perform data transformation. We use the internal Weka implementation for min-max normalization for both neural networks and *k*-NN.

##### Practical Considerations for Pre-Processing

To facilitate the pre-processing step we refer the reader to some popular data mining toolboxes/libraries including R, MATLAB, Octave, Weka, RapidMiner, Rattle GUI, NumPy/Scipy, scikit-learn, ELKI [179], *etc*.

### 4.5. Data Mining

Section 3.4 introduced the data mining step of the KD process. This section illustrates in more detail how the data mining step of the KD process (see Section 3.4) should be performed.

#### 4.5.1. Generic Data Mining Process

**Figure 7:**
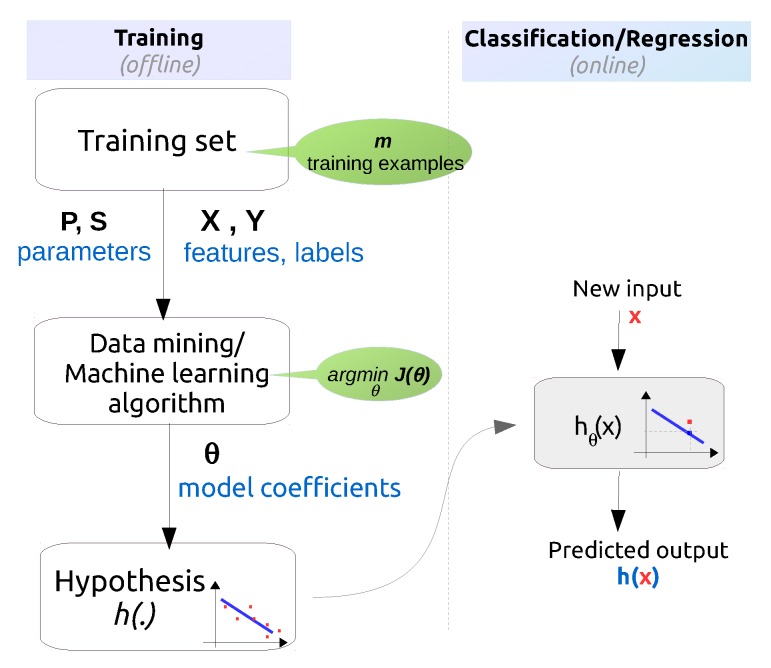
The supervised learning process: training phase (**Left**) and classification/regression (**Right**).

Figure 7 presents the data mining/machine learning process. On the left hand side of the figure, the training phase is illustrated while on the right hand side, the classification/regression is performed. Most systems perform the training offline on batch data (a static dataset that is available rather than on streaming data) and are only afterwards used online (often on streams of data) for the classification/regression task.

The training dataset consists of a set of feature vectors and label vectors. The mathematical representations of the feature vector and label vector are:

(4) $$\begin{array}{ccc} x={\left[\begin{array}{c} x_{0},x_{1},\cdots ,x_{n} \end{array}\right]}^{T}\in R^{n+1} & & y\in R \end{array}$$

A pair (x, y) represents a training example or instance for the algorithm. Several such training examples are used by the learning algorithm to build a model. The values of the features and labels can be real-valued numbers, discrete-valued numbers or categorical.

Our hypothesis about the stochastic process *f* to be learned (approximated) is denoted by *h* [180]. By $h_{\theta }\left(x\right)$ in Figure 7 we refer to a hypothesis with coefficients *θ* learned using a set of tuning system parameters, *S*, and model parameters (which can be *internal* or *general* parameters- explained below), *M* (e.g., S=*[window size]*, M=*[number of hidden layers in NN, size of the hidden layers, α, λ]*, *etc*.). The coefficients, *θ*, are learned through an iterative update process performed by an internal optimization algorithm of the learning algorithm itself. During this process the coefficients *θ* are optimized by minimizing a *cost* or *error* function with regard to *θ*, denoted as J(θ). This process can be controlled by tuning internal parameters *α* and *λ*, where *α*- the *learning rate*, controls how fast the algorithm converges to the optimal solution for *θ*, while *λ*- the *regularization parameter*, controls the complexity of the resulting model in order to avoid *over-fitting* (see Section 4.6). The process of learning the coefficients *θ* can be additionally controlled by tuning the *general* model parameters such as the number of nodes in the hidden layer of the neural networks learning algorithm.

The learning algorithm picks the hypothesis, $h_{\theta }\left(x\right)$, that is the best guess for *f* given the set of tuning parameters *P* and *S*. $h_{\theta }\left(x\right)$, sometimes simply denoted as h(x), is computed based on a set of *m* training examples, called the *training set*, denoted as *i.e*., $\left\{\left(x^{\left(1\right)},y^{\left(1\right)}\right),\left(x^{\left(2\right)},y^{\left(2\right)}\right),...,\left(x^{\left(m\right)},y^{\left(m\right)}\right)\right\}$. Usually, there is a trade-off between the number of training examples needed for a learner to converge to a highly accurate model, also considered as sample complexity, and the amount of resources it requires to construct such model, also considered as computational complexity [181].

The learned hypothesis function is used to predict outcomes for new, previously unseen inputs, *i.e*., to map features *x* to values *f(x)*. Section 2.2 already discussed a number of frequently used learning algorithms. One major challenge is how to select the right learning algorithm that will solve a target problem? There are no rules of thumb for selecting a concrete learning/mining algorithm, but in practice the following approach is used:

1. Start with a simple algorithm which is easy to understand and easy to implement.
2. Train the algorithm and evaluate its performance.
3. If the algorithm performs poorly, try to understand why:
  - Diagnose if the model is too complex or too simple, *i.e*., whether it suffers from *under*- or *over-fitting*.
  - Depending on the diagnosis tune appropriate parameters (system parameters, model parameters, *etc*.).
  - If necessary analyze manually where and why errors occur (*i.e*., error analysis). This helps to get an intuition whether the features are representative.
4. If the algorithm still performs poor, select a new one and repeat all the steps.

According to [182], some of the most popular and influential data mining algorithms in the research community (able to solve problems like classification, clustering, *etc*.) are: *C4.5*, *k-Means*, *SVM*, *Naive Bayes*, *k-NN*, *etc*. Selecting the most suitable algorithm will require iterations with the next step of the KD process, thus we leave the details about model selection and performance optimization for the next section.

#### 4.5.2. Mining the Device Fingerprinting Problem

For the device fingerprinting classification problem we selected the following four classification techniques: *k*-NN, decision trees, logistic regression and neural networks (see Section 2.2 for a brief description of each). We used the Weka data mining toolbox for training. The learning algorithms we selected from the Weka GUI are the following: for *k*-NN the implementation from inside the class of “lazy” algorithms called *IBk* (Instance Based learning with parameter k) with parameters: K=1 neighbours; for decision trees the C4.5 implementation from inside the “trees” class called *J48* with parameters: confidencefactor=0.25 for pruning, M=2 as minimum number of instances per leaf; for logistic regression the implementation from inside the "functions" category called Logistic with parameters: $\lambda =10^{-8}$ and for neural networks the implementation from inside the “functions” category called MultilayerPerceptron with parameters: epochs=500, hiddenlayer=1, sigmoid nodes in hidden layer =10, α=0.3, momentum=0.2. As such, it is clear that for the purpose of this tutorial we selected very simple and basic algorithms containing few configurable parameters but also more complex ones such as neural networks with up to five configurable parameters.

Besides the model parameters, we need to decide on the target system parameters. More specifically, we need to decide on the number of IAT data points that will be used to create training examples (*i.e*., histogram instances with *N* feature bins). We introduce this system parameter as the Window size, *W*. In order to select the optimal number for *W*, we perform the following test: we select several candidates for *W* (2000, 5000, 7000, 10,000, 12,000 and 15,000) and evaluate the performance of each classifier (using cross-validation) for device type classification trained on the resulting $\frac{Size}{W}$ per device training examples, where Size is the total number of IAT data points per device trace. This resulted in having more training examples for each device. At the end, we select the number of *W* that leads to the minimum prediction error. Best results showed W=5000 with 4933 adequately labeled training examples in total. This test is similar to the test performed in Section 4.4.2. Another option to determine the optimal Window size may be to calculate the closeness of the distributions of each training example (e.g., separately for all training examples derived from DN1 trace) to the distribution of the corresponding original full data (e.g., full DN1 trace) by means of statistical similarity measures such as coefficient of correlation and covariance and take the average. Repeat the test for several values of *W* and select *W* that leads to the best closeness score.

We train the algorithms, separately for device and device type classification, with the dataset prepared in the pre-processing step of the KP process described in Section 4.4. The native file format for the input dataset is an ASCII text file called ARFF (Attribute-Relation File Format), which was developed for use in Weka. The file describes a list of instances that have a common set of features/attributes. However, Weka has also the capability to read flat CSV format files, where the convention is as follows: first row contains feature/attribute names followed by the name for the label vectors (all separated by commas), each following row represents a training example with features/label values listed in the same order (also separated by commas). We use the CSV format for the dataset, where the first row contains feature names having the format Bi, where i=[1,N] corresponds to each of the N features (*i.e*., histogram bins), and the label vectors named simply as class. The resulting dataset can be seen as a m×(N+1) matrix (without the first naming row).

##### Practical Considerations for Data Mining

Several data mining toolboxes/libraries provide implementations for the machine learning algorithms introduced in Section 2 and beyond, such as: Weka, RapidMiner, Orange, KNIME, Rattle GUI, ELKI, Java libraries such as Vowpal Wabbit, Shogun, Python libraries including scikit-learn, libsvm, Pybrain, TensorFlow for deep learning, *etc*.

### 4.6. Performance Evaluation

Section 3.5 introduced the performance evaluation step of the KD process. This section goes into more details to clarify how this step should be performed.

#### 4.6.1. Performance Evaluation Techniques

**Figure 8:**
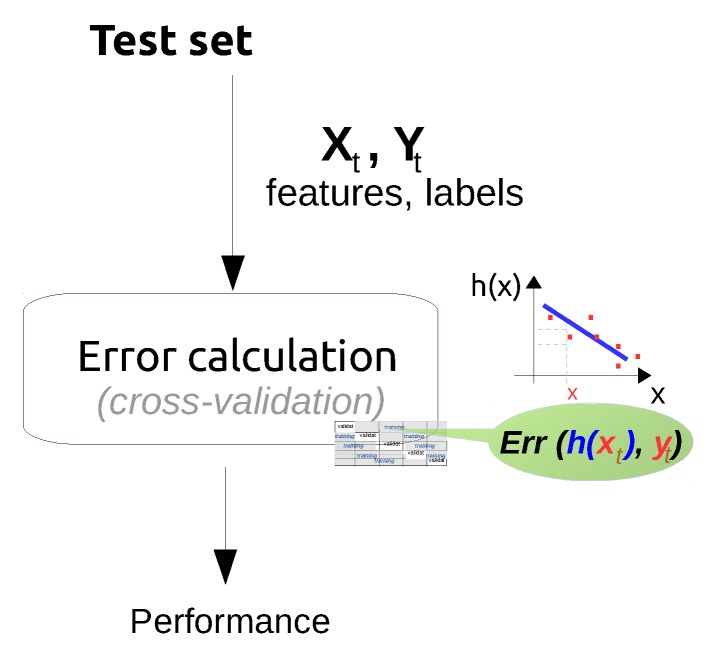
Testing the correctness of a model after the learning phase using a separate test dataset.

**Figure 9:**
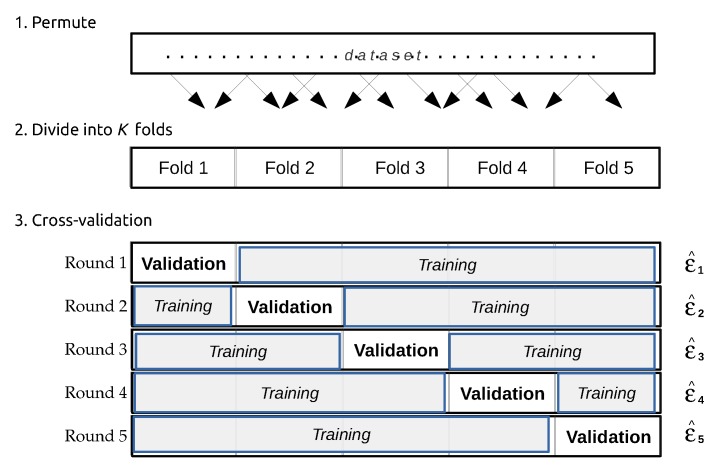
Illustration of a *5*-fold cross validation process. The general dataset is permuted and split into five folds. Afterwards, cross-validation is run through five rounds. In each round, one of the folds is kept for testing while the others are used for training.

The overall performance evaluation process is summarized in Figure 8. For testing purposes, a separate test set, to which the learning algorithm was not exposed at training time, is used to evaluate the performance of the hypothesis, h(x), trained in the previous step. This is often done through *k-fold cross-validation* as illustrated in Figure 9 for the case of *k = 5*.

The general dataset is first permuted and split into five folds. Then, cross-validation is run through five rounds. In each round, one of the folds is kept for testing while the others are used for training. The test error, $\epsilon_{i}$, is calculated and used as an estimate of the prediction error for that round. At the end, the average error over all folds is computed as: $\hat{\epsilon }=\frac{1}{k}\sum_{i=1}^{k}{\hat{\epsilon }}_{i}$.

In practical situations the case for *k = 10*, *i.e*., *10-fold cross-validation*, has become the standard way of measuring the prediction error of a learning algorithm. Although, several tests and theoretical evidence proved the number 10 to be the right choice for getting reliable prediction error estimates, data mining and machine learning circles still debate about the best choice for the number of *k* [183]. For more reliable estimates of the prediction error, the author of [184] recommends to use stratified 10-fold cross validation, an approach where prior to cross validation the data needs to be arranged so that in every fold, each class comprises approximately the same number of instances. Individual 10-fold cross-validation runs generate different results due to randomizing data instances before selecting the folds. As such, another improvement proposed by [185] is to repeat 10-fold cross-validation 10 times, *i.e*., 10×10 cross validation, and take the average over the individual results.

#### 4.6.2. Performance Evaluation Metrics

**Figure 10:**
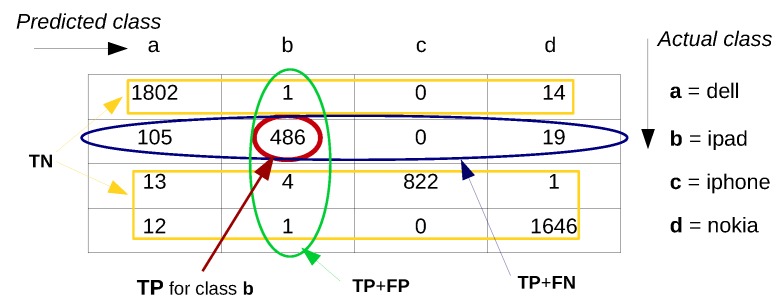
Confusion matrix interpretation for class *b*.

Figure 8 also shows that an adequate error metric has to be selected to estimate the prediction or *generalization* error that the model will tend to make on future unseen examples. As mentioned in Section 3.5, for a classification task the confusion matrix and metrics derived based on it are commonly used. Figure 10 depicts a typical confusion matrix where the rows indicate the actual class an instance belongs to, while the columns indicate to which class it was assigned to by the prediction algorithm. For instance, it can be seen that items from class *b* have been correctly classified 486 times (*i.e*., TP—true positives) and have been classified as class *a* and *d* 124 times (*i.e*., FN—false negatives). At the same time, one instance of class *a*, four instances from class *c* and one instance from class *d* have been classified as belonging to class *b*, thus representing FP—false positives. All the other cells of the matrix represent the TN—true negatives.

Four widely used metrics are computed based on the confusion matrix: accuracy, precision, recall and the F1 score.

- The accuracy of a classification system is defined as $accuracy=\frac{TP+TN}{TP+TN+FP+FN}$, where TP, TN, FP and FN are respectively the: *true positives*— instances that are correctly classified as the actual class, *true negatives*—instances that are correctly classified as not being the actual class, *false positives* or *Type I* error—instances that are misclassified as the actual class, and *false negatives* or *Type II* error—instances from the actual class that are misclassified as another class [169]. In machine learning and data mining literature the accuracy is also referred to as the overall recognition rate of the classifier [169] and gives information about the percentage of correctly classified instances.
- *Precision*, defined as $precision=\frac{TP}{TP+FP}$ represents the fraction of correctly classified instances within all instances that were classified (correctly and incorrectly) as a particular class. In other words, it is the percentage of positive instances within all positive labeled instances. Hence, it can be thought as the exactness of the classifier.
- *Recall*, *sensitivity* or the true positive rate, defined as $recall=\frac{TP}{TP+FN}$ is the fraction of correctly classified instances of a particular class within all instances that belong to that class.
- An alternative way of using precision and recall is to combine them into *F1* or *F-score* defined as the *harmonic mean* of *precision* and *recall*: $F1=\frac{2\times precision\times recall}{precision+recall}$.

In case of imbalanced classes, the last three metrics (precision, recall and the F1 score) give more precise results of a classifier’s performance. For instance, if we have 98% instances of class A, and only 2% of class B, an accuracy rate of 98% is not an adequate measure, because it cannot indicate whether the classifier correctly classifies only the instances of class A and misclassifies all instances of B. For this purpose, precision and recall are used. In general, a good classifier should have high precision and high recall. For some applications it is important to be able to control the recall and precision values, for example when it is more important to reduce the false negatives rate (e.g., in predicting whether a patient has cancer) than the false positive rate. Such scenarios require a recall-precision trade-off.

The baseline any learning algorithm should be compared against is random guessing. If the algorithm performs the same or worse than random guessing, then no learning has been realized. Similarly, algorithms that have 100% precision and recall most likely are the result of a fault in one of the KD steps. When evaluating several algorithms at once, their performance should be ranked against the baseline and against each other.

#### 4.6.3. Improving the Performance of a Learning Algorithm

**Figure 11:**
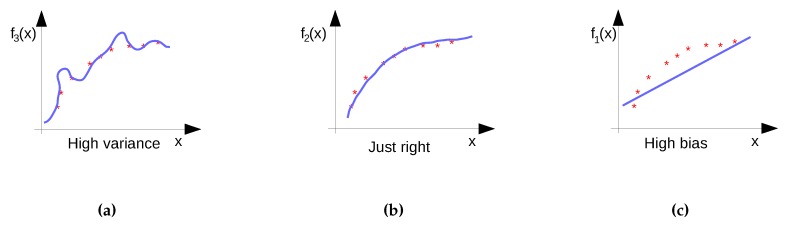
Explanation of bias-variance problem (**a**) High variance; (**b**) Optimally trained model; (**c**) High bias.

The previous paragraphs discussed methods and metrics to evaluate the performance of a learning algorithm. This section will discuss how to improve the performance of learning algorithms, which in practice requires a two-step process (i) diagnose why a learning algorithm performs poor, and (ii) apply the correct set of actions to improve its performance. To be able to perform correct diagnosis, it is important to understand that one of the main reasons preventing a learning algorithm to perform well on new unseen examples is the bias-variance problem. In order to understand the bias-variance problem, first, it is important to note that in machine learning, more complex models are not always better at predicting or classifying. Complex models tend to "over-fit" the training data (*i.e*., over-fitting occurs), meaning that the model is trained to match the dataset too much, thereby modeling not only the underlying relationships but also the random error or noise that is present in the data. Models that over-fit the data are said to have a high variance problem. Figure 11a shows an examples of a model with high variance. Such models are too complex and it is to be expected that the model will appear to perform less well at predicting on new unseen data than on the original dataset used for training. In machine learning and data mining, this situation is known as that a model fails to generalize well. In contrast, simple models, as one shown on Figure 11c, tend to "under-fit" the training dataset (*i.e*., under-fitting occurs), meaning that the statistical model is too simple with regards to the data it is trying to model and as such fails to model crucial underlying particularities of the dataset. As indicated on Figure 11c, models that under-fit the data are said to have a high bias problem.

How to know when a model has struck the right balance and is not under-fitting or over-fitting the data it seeks to model? Figure 11b shows an examples of an optimally trained model, *i.e*., that is accurate and expected to generalize well.

The generalization ability of a model can be tested by diagnosing whether it suffers from high bias or high variance. One approach to diagnose the bias-variance problem is using learning curves [180]. The idea behind learning curves is to observe the test or cross-validation (CV) error and the training error at the same time with respect to a parameter that represents a measure of varying amounts of learning effort [186]. The training error is the estimated error that the model makes against the same dataset on which is was trained. As such, it will not be a reliable estimate of the prediction error that the model will make on future unseen examples, but is a useful measure for diagnosing the bias-variance problem as is further explained.

**Figure 12:**
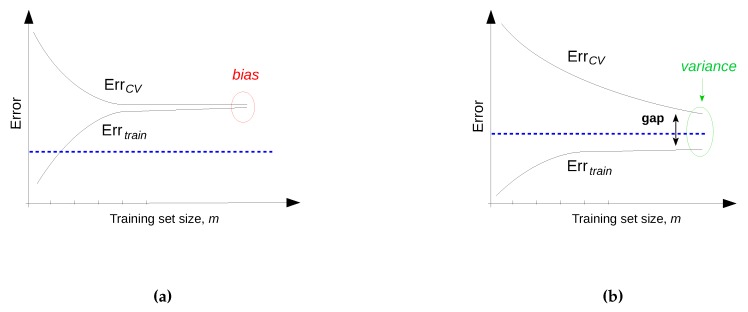
Diagnosing the bias-variance problem (**a**) Learning curves indicating high bias; (**b**) Learning curves indicating high variance.

Typically, learning curves are plots of the training and the cross-validation error as a function of the number of training examples or the number of iterations used for training. Figure 12 shows two characteristic learning curve shapes when plotting the training and the validation error as a function of the training set size, *m*. The bias is highlighted with red, the variance with green, while the blue is the desired prediction error. In case of high bias (Figure 12a) the model is over-fitting and as such increasing the number of training examples causes the training and the validation error to converge to an unacceptably high error (above the desired error). In contrast, in case of high variance problems (Figure 12b) the training error stays below the acceptable value while the testing error tends to be much higher. In this case, adding more training data can help to decrease the validation error closer to the desired value. The intuition behind the phenomena in Figure 12 is that for small values of *m* it is easy to fit the training data, which is why the training error $Err_{train}$ is small, but the produced model will not generalize well, which is why the test error $Err_{CV}$ is high. By increasing the training set size *m*, it gets more difficult to fit the data perfectly but the model tends to generalize better to new instances, which is why the training error increases, while the test error decreases with *m*. However, for even higher values of *m*, in case of high bias both training and testing error do not satisfy the desired error threshold (blue line), while in case of over-fitting both errors seem to approach the desired value, and typically a gap appears between them as illustrated on Figure 12b.

**Figure 13:**
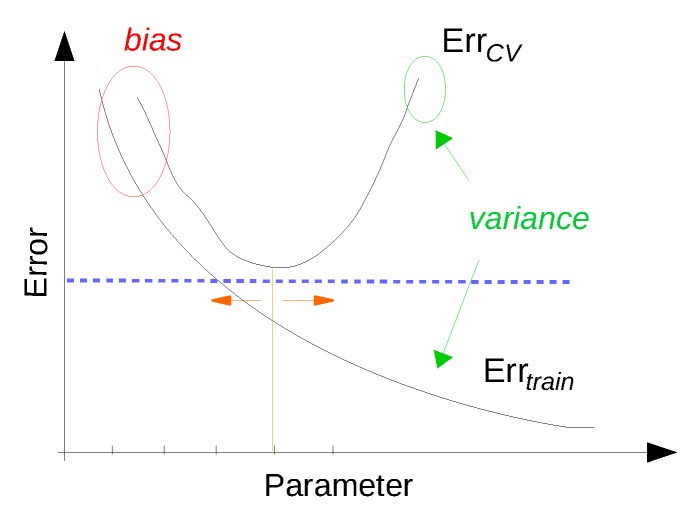
Tuning a learning algorithm.

Additionally, the bias-variance problem can also be solved by controlling the complexity of the trained model through tweaking its internal and general parameters. Typically, the regularization parameter *λ* [123] is controlled as an internal parameter for parametric models. Examples, of general model parameters that can be tweaked are: the number of neighbours in k-NN classification, the number of hidden layers or the number of sigmoid nodes in the hidden layer of neural networks, *etc*. Choosing a simple model (e.g., a neural network with two nodes in the hidden layer) will increase the risk of having high bias, because a simple model tends to under-fit the training data, leading to high training and high validation error. Choosing a too complex model (e.g., a neural network with 100 nodes in the hidden layer) increases the risk of having a high variance problem, because a complex model tends to fit the noise in the training data and does not generalize well, leading to high validation error but small training error. To decide on optimal configurations, it is helpful to plot the test and (cross)validation error with regard to the model parameters as shown in Figure 13. The optimal choice with the minimal validation error is denoted in dotted blue.

Finally, another configuration parameter that can cause a learning algorithm to perform poorly is related to the convergence of the internal optimization algorithm used by the learning algorithm itself during the training phase, *i.e*., the rate with which the algorithm stabilizes. Typically, gradient descent is the internal algorithm integrated into a machine learning algorithm and is used for optimizing the model coefficients. Often, the convergence can be achieved by increasing the number of training iterations, also known as epochs, that the algorithm is allowed to run. One epoch corresponds to one update step when calculating/optimizing the model coefficients during training phase. It is one complete sweep over the training data. Using too few epochs results in inaccurate models. Another tunable parameter influencing the convergence rate is the learning rate *α*, that was introduced in Section 4.5.

To summarize, the goal of tuning a learning algorithm, also referred to as model selection, is to avoid bias and variance while keeping the test error ($Err_{CV}$) below the desired prediction error. Table 9 summarizes the discussed actions one can take to improve the performance of learning algorithms based on the diagnosed problem.

#### 4.6.4. Performance Evaluation for the Fingerprinting Problem

For the device fingerprinting classification problem we will evaluate the performance for the data mining algorithms that were selected in the previous step: *k*-NN, decision trees, logistic regression and neural networks. We use the 10-fold cross-validation implementation available in the Weka toolbox to obtain performance results of each model. As was anticipated in Section 4.2 the GaTech dataset is quite unbalanced and as such per-class performance evaluations should be used. Thus, we present results in form of a confusion matrix and derive per-class metrics such as precision and recall from it. We start our discussion by presenting partial results for the device classification problem (*i.e*., identify the exact device ID), and then demonstrate detailed results, analysis and evaluation procedures for the device type classification problem (*i.e*., identify the device type). By intention we select the default parameters for the neural networks algorithm in Weka, to demonstrate how the recommended actions proposed in Section 4.6.3 can be applied to improve a learning algorithms’ performance.

Table 10 presents the confusion matrix for the *k*-NN learning algorithm trained for *device classification*. The model performs generally well, as the majority of the predictions are located in the diagonal elements of the confusion matrix, which corresponds to correctly classified instances. For instance, for the *dell1* class 164 instances were correctly classified as *dell1*, for *dell2* 190 instances were correctly classified as *dell2*, *etc*. By dividing the sum of the diagonal elements (*i.e*., 4033) with the total number of instances (*i.e*., 4926) we obtain the rate of correctly classified instances of the model, *i.e*., ∼82%. It can be seen that the remaining ∼18% misclassified instances occurred mostly because of confusing *dell2* with *dell3*, *dell4* with *dell5*, *ipad1* with *ipad2* and *dell2*, and *nokia1* with *nokia2*, which was already hinted at by high $R^{2}$ scores in Table 5 of Section 4.3.

Similar conclusions can be derived from the results of the remaining algorithms, available at [188]. Decision trees demonstrated the best performance with 91% correctly classified instances, while logistic regression achieved 88%. Only neural networks demonstrated poor performance, *i.e*., 25%, which was not expected from the data analysis $R^{2}$ results. However, it turned out that neural networks showed similarly poor performance for the device type classification problem, hence, the performance of neural networks will be discussed in more detail in the subsequent part of this section. The goal is to exemplify how to diagnose whether a algorithm can be improved or it should be discarded in the model selection phase.

Table 11 presents the summarized confusion matrix, precision and recall and their weighted average values over all classes for: *k*-NN, decision trees, logistic regression and neural networks learning algorithms that are trained for device type classification.

Table 11 shows that the *k*-NN trained model performs generally well, confusing mostly *ipad*s with *dell*s and *dell*s with *nokia*s, as anticipated by the high $R^{2}$ scores, especially for *dell* and *nokia*, obtained in Section 4.3. The highest precision is for the *iphone* class, *i.e*., 1, as no instance from other classes were confused *iphone*s (FP=0), followed by *ipad* and *nokia* with a precision score of 0.98 and *dell* with precision 0.935. The highest recall was obtained for *nokia*, *i.e*., 0.996, followed by *dell*, *iphone* and *ipad* with scores 0.993, 0.979 and 0.784, respectively.

For decision trees, Table 11 shows also very good results, misclassifying only a few instances. As it can be seen, both precision and recall are above 0.99 for all classes, meaning that the model is resistant to false positive and false negative errors and this learning algorithm was able to capture differences between device type classes based on the training instances better then *k*-NN.

The logistic regression model performs slightly worse then *k*-NN and decision trees, having more classification errors and confusing also classes that had lower $R^{2}$ scores like *iphone*s with *nokia*s. The precision and recall are slightly lower then in previous models, having average precision and recall both of 95.3%, which is ∼4% lower then average precision and recall of decision trees, and ∼1% lower then in case of *k*-NN.

The performance of the neural networks is significantly poorer compared to the other models, confusing many more *dell*s with *nokia*s (and vice versa), and *ipad*s with *iphone*s as was anticipated by high $R^{2}$ scores obtained in Section 4.3, but also *iphone*s with *dell*s which had lower $R^{2}$ scores. The precision and recall scores are drastically lower for each class, having an average precision scores of 47.9% and recall scores of 46.2%, which is at least 48% lower then average precision and recall of previous models. This is suspicious as the performance of neural networks should be comparable or better than decision trees [189]. The main advantage that neural networks have over decision trees is their flexibility to train simple to very complex models through tuning a large parameters space they have (recall that neural networks are known to be universal approximators!). As a result, we have to look into the details of the mining algorithm and diagnose what is going wrong.

As discussed earlier, to gain insight in the behavior of an algorithm, besides the validation error we also need information about the training error. For this purpose, we run validation of neural networks in Weka again using the training set instead of cross-validation for testing, to obtain the training error. The training error turned out to be as high as the cross-validation error. As discussed, high training errors in combination with high cross-validation errors might indicate the existence of a high bias problem. According to the guidelines from Table 9, increasing the complexity of the algorithm (in this case the number of sigmoid nodes in the hidden layer) might help decrease the cross validation error. Hence, an option is to calculate the training and cross-validation error again for more complex models, e.g., 15, 20, 50 nodes and plot them similar to Figure 13. Unfortunately, the outcomes showed that this is not likely to solve the problem, and that high error rates persist. Similarly, the classification errors are also unacceptably high for simpler models: 1, 2, 3, 5 and 6 nodes in the hidden layer. Returning to Table 9, a high bias problem can also be solved by either: reducing the number of training instances, adding more features or decreasing regularization. The first option did not influence the accuracy of the model but it does improve the training speed, which is practical for computationally expensive algorithms. Adding more features might solve the problem. However, our model already considers a considerable number of features, *i.e*., the 500 bins of the histograms, therefore adding more features will likely not have a big influence on the performance. The last option is also not a relevant candidate as no regularization was used.

**Figure 14:**
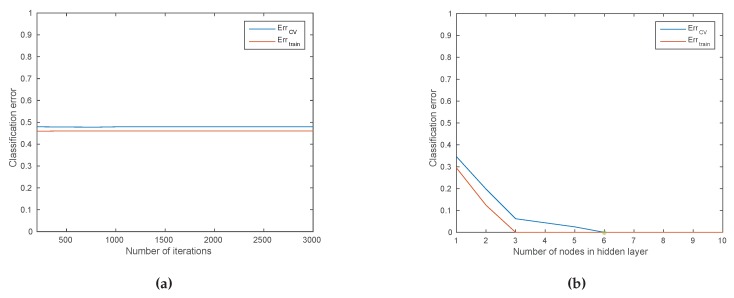
Tuning the performance of neural networks: solving the convergence problem. (**a**) Learning curves, α=0.3, HLsigmoid=2; (**b**) Model selection, α=0.1, 500 epochs.

Returning to Table 9, we see that there is a third issue that can prevent a model from performing well, namely the *convergence problem*, which can be solved by using more training iterations or reducing the learning rate. (i) To evaluate the influence of training iterations, we need to plot the learning curves comparing the performance to the number of training iterations, *i.e*., epochs. For this purpose, we calculate the training and validation error for several input values of number of epochs: 200, 500, 800, 1000, *etc*. In order to keep the diagnosis process time-efficient, we selected a simpler model with one hidden layer with two sigmoid nodes, HLsigmoid=2, whereas all remaining parameters are the same. Figure 14a shows the corresponding learning curves. As it can be seen, both the training and the test error are relatively high for all cases, meaning that increasing the training iterations does not solve the problem. (ii) Alternatively, a last possible issue could be the use of a learning rate *α* that is too high causing the algorithm to fail to converge on the best solution. Namely, with a higher learning rate the algorithm uses bigger steps during its convergence process. If the step is to big, the algorithm will likely never approach the optimal solution. In order to verify this option, we recalculated the train and validation error for the same parameter set (HLsigmoid=2, epochs=500) but for the value of α=0.1. The results showed that both training and validation error decreased below 20%. Hence, tuning the learning rate solved the convergence problem.

Now that the convergence problem is solved, we can re-iterate the previous processes to further fine-tune the parameters of the learning algorithm. For this purpose, we calculated the training and validation error one last time with regard to the number of sigmoid nodes in the hidden layer of neural networks, *i.e*., the model complexity. Figure 14b shows how the errors vary with the model complexity (HLsigmoid=1,2,3,5,6,10). The best model was one with 6 hidden nodes. Increasing the complexity of the model further may introduce over-fitting, and makes no sense since the current 6 sigmoid configuration has optimal training and validation errors.

Table 12 presents the confusion matrix for the new neural networks model. Although the neural network model initially seemed to perform worst out of all investigated algorithms, with the optimized configuration settings the model now classifies correctly instances from all classes.

##### **Practical Considerations for Performance Evaluation** 

The models trained in the previous KD step can be evaluated using some of the following data mining toolboxes/libraries: Weka, RapidMiner, Orange, KNIME, Rattle GUI, ELKI, Vowpal Wabbit, Shogun, scikit-learn, libsvm, Pybrain, *etc*.

### 4.7. Using the Discovered Knowledge

As discussed in Section 3.6, the last KD step deploys and integrates the learned models within an operational system. Typically, the previous steps of the knowledge discovery framework were realized using an available data mining toolbox offline which is not suited for a product environment. To translate this knowledge into a production solution, the experimenter needs to have more understanding of the internal learning algorithm that was trained and implement the trained model on the target system.

During this process, the programmer will have to investigate additional operational aspects such as: how to collect the data, how to extract the features from the data, how much data has to be collected as input for the prediction model, pre-processing of the data, *etc*.

However, in this paper we exemplified and implemented the first five steps of the knowledge discovery framework. The sixth step requires developing specific algorithms which can use inferences of the designed model within the target production environment. As there is no generic methodology for this step, it is out of scope of the purpose of a generic educational tutorial on designing data-driven wireless communication solutions. Below, we attempt to formalize the steps that need to be taken to implement the model that was designed through the previous five KD steps, towards having a production ready system for real-time detection of malicious devices.

#### Using the Discovered Knowledge for the Fingerprinting Problem

In order to implement the neural network model selected in previous step, we identify several steps that need to be realized.

1. Extract the model coefficients, *i.e*., the weights between each pair of neurons in the network, calculated by Weka. The trained neural networks model consists of three layers: the output, hidden and input layer. Each layer consists of a set of sigmoid nodes. The Weka GUI outputs: (i) the output layer sigmoid nodes (4 for device type classification or 14 nodes for device classification), which are the output connections to each class, and their weights towards each node in the hidden layer, (ii) the hidden layer sigmoid nodes (6 nodes) and their weights towards each node of the input layer. Those coefficients will act as parameters within a data mining library. Because the target system for wireless device fingerprinting can be a general purpose computer located in the corporate access network, Weka’s Java API may be a candidate for model implementation (the only prerequisite is the JVM installed).
2. Define how to collect data in the online system. Packets can be captured using tcpdump [190], or any other traffic-capture mechanism and packet analyzer.
3. Define how to extract the inter-arrival times (IAT), *i.e*., the difference between arrival times of two successive packets, produced by the same device or device type class and the same application running on top of that device. This may be achieved by processing the captured traffic flows, *i.e*., sequence of packets with same source and destination IP, source and destination UDP/TCP ports.
4. Define how to pre-process the raw captured data. Histograms should be created similar to how the training and testing data was pre-processed in Section 4.4.2.
5. Define how many packets (*L*) to collect to form the set of features needed to feed into the classification model and perform a prediction task. This is defined by the previously established system parameter ‘window size’ *W* used to train the model. *W* determines the length of the traffic flow, *L*, which is the number of packets that need to be collected to form the histogram with 500 bins (features) and feed as a new instance into the classifier. The relation between *W* and *L* is L=W+1.
6. Deploy and start the classification engine on the target system.

### 4.8. Final Considerations: Future Recommendations and Implementation Challenges

Table 13 provides a structured overview of the knowledge discovery process that was exemplified in the Case study of Section 4. The first two columns of Table 13 present the knowledge discovery steps and sub-steps, respectively; column “I: Custom approach” summarizes the approach per KD step that was taken to solve the wireless device fingerprinting problem in the work of Radhakrishnan *et al*. [12], while column “II: Proposed methodology” a solution to the same problem that utilizes best practices from the data mining community. Finally, column “Recommendations” provides general conclusions by comparing the custom approach, [12], with the approach that uses the proposed methodology. This column summarizes general recommendations for a successful data science application to any problem in the field of wireless networks research. Below we discuss differences between both approaches for each KD step, discuss pro/cons and give general recommendations.

#### ***Understanding the domain*** 

In the first step, the problem was studied and the hypothesis was stated. While Section 4.2 gives a brief introduction on the selected problem, a more in depth study can be found in the original work of Radhakrishnan *et al*. [12]. Both approaches, I and II, correctly translated the problem into a data science classification problem. In general, we recommend the novice to consult Section 2 to properly pursue this step. Then, the data collection requirements were identified using domain knowledge, which resulted in a set of IAT traces from several wireless devices. Generally, real (traces generated from real wireless network deployments or experimental facilities) and synthetic data (data generated through simulations) are used in practice to validate the hypothesis and design the prediction model. Due to the inherent characteristics of wireless networks that cannot be reflected through simulations or mathematical models, recommended is to collect experimental data from real-world wireless networks. This might be a complex, costly, and time consuming approach; however, real traces offer a more realistic understanding of the actual system, compared to synthetic data which may have difficulties to accurately capture the underlying particularities of wireless networks. Ultimately, data is at the center of data-driven research, if the data is not of good quality nor sufficient, (valid) knowledge cannot be extracted.

#### ***Understanding the Data*** 

After the analysis in KD step 1, it should be known how the data can be used to verify the stated hypothesis and also validate if the selected dataset can solve the problem. The actual implementation of these validation techniques is done in the Understanding the data step. For this purpose, the custom approach exploited visualization techniques such as PDF and average IAT-values plots. Although this might clarify whether the collected data can be used to classify devices based on their IAT patterns it does not give sufficient information about the data quality nor about the need for subsequent data cleaning. The proposed methodology suggests to complement visual techniques with computational techniques. Hence, the proposed methodology used the 5 number summary to get more insight in the data and identify potential outliers and explore data transformation requirements; in general to identify data pre-processing requirements. Finally, the $R^{2}$ was used to reveal the predictive power of one trace against others. In general, for this step it is recommended to combine visual and computational techniques, that were introduced in Section 4.3.

#### ***Data Pre-Processing*** 

After the data pre-processing requirements have been identified, data has to be cleaned, transformed and/or reduced, through the *Data pre-processing* step. Both approaches used histograms as a data reduction and feature extraction technique. However, the custom approach missed two important steps: data cleaning and transformation. This can be explained by the absence of the data validation step that should have been taken prior. In fact, it is not clear how the PDF plots in step 1 of approach I, [12], have been obtained without any additional pre-processing steps such as: histogram adjustment and outlier removal that have been applied in approach II. Furthermore, there is no clarification on the optimum number of selected histogram bins for training data representation. Recommended is to perform all three data pre-processing steps when needed, in order to obtain the optimum training dataset that will enhance prediction accuracy and efficiency. The output of this step should be the optimal training set representation.

#### ***Data Mining*** 

Finally, after the training set has been created *Data mining* is performed. Approach II used the created training dataset for training several machine learning algorithms including *k*-NN, NN, decision trees and logistic regression. Then the best model was selected as measured according to a performance metric (*i.e*., precision and recall) and a model selection algorithm (*i.e*., cross-validation). The custom approach I considered only one algorithm (NN) without justification on the algorithm selection. Taking only one model is not in accordance with best practices in data science, as other models might perform better in terms of accuracy and/or computational efficiency.

#### ***Performance Evaluation*** 

The trained models are evaluated in the Performance evaluation step using a separate dataset. Typically, due to insufficient data resources, the original dataset is split into a test and training set, which can be automatised through the cross-validation algorithm. Finally, the performance is measured by standard metrics such as: precision, recall, accuracy and F-score (for a classification task!), and presented in a standard way using a confusion matrix. However, approach I uses a custom approach for extracting the test set and presents only partial performance results for a small number of devices. Also only accuracy and precision were selected as the performance evaluation metrics. We recommend to use best practices established in the data mining community for the target problem type, as examined in Section 4.6. For instance, the proposed approach II used the confusion matrix to show complete results for all considered classes of devices. From this representation, all remaining performance metrics can be extracted.

#### ***Using the Discovered Knowledge*** 

In the final stage, when the best model has been found, it has to be elaborated how the model will perform within a real system, and what the practical limitations are towards its integration in an operational environment. Practical aspects about Using the discovered knowledge are discussed. As this step is problem specific it is not feasible to give general recommendations for it. However, in Section 4.7 we depicted the necessary steps towards a real-life model implementation taking into account generic aspects that are common to most implementations such as: how to deploy the model, how to collect the data, how to extract features, how to pre-process data, *etc*. Such details were also missing in the original work in [12].

**Implementation evaluation methods:** In general, the most common approaches to evaluate the proposed model in terms of implementation, *i.e*., to estimate its performance in a future fully operational system, are: analytical modeling, simulation and experimental validation.

Analytical modeling is a complex implementation technique that typically assumes certain approximations and assumptions to obtain a mathematical closed form solutions to evaluate the performance of the proposed scheme. However, analytical models may lead to imprecise results with limited confidence especially in the context of real wireless networks.

Simulation is the most popular model implementation evaluation technique because of its low cost and time of realization. In particular, first a certain wireless network scenario has to be simulated (e.g., generate a number of interference and traffic patterns, create a propagation model of the wireless medium, *etc*.), then the designed prediction model is added to the system to analyze and make predictions on the target data (e.g., RSSI, LQI, PRR, *etc*.), and ultimately improve the performance of the wireless network (e.g., increase throughput, minimize latency, optimize energy consumption, *etc*.). In fact, most of the data science applications in wireless network research analyzed in Section 2 use real data for modeling (*i.e*., KD steps 1-5), generated from a wireless test facility or obtained from an online repository, while simulations for real-deployment evaluation (*i.e*., KD step 6). However, simulations have its disadvantages and limitations compared to experimental evaluations due to (i) difficulties to accurately model the underlying wireless transmission properties such as channel characteristics and antenna diversity, and (ii) difficulties to simulate the hardware's imperfections and dissimilarities between devices of the same type, which often have a considerable impact on the overall network performance [191]. Finally, results can vary when executing identical scenarios on different wireless network simulators.

Hence, for wireless networks research experimental validation is necessary to complement results from simulations. Experimental validation refers to the process of evaluating the performance of the modeled system behavior in a real system deployment. It has gained more attention recently due to the (i) rise of available infrastructures with heterogeneous wireless devices and technologies provided for experimentation purposes, and (ii) increased degree of automation for controlling and configuring these devices. Finally, experimental model validation can provide the most dependable results.

**Implementation challenges:** Data science approaches have shown promising results and increasingly intensive adoption for improving the performance and solving various problems of wireless networks. However, there are a few challenges and limitations that should be considered when implementing the extracted patterns in real wireless networks: (i) limitations due to resource constrained wireless devices, e.g., limited battery and computing power, little memory, *etc*.; and (ii) limitations due to constrained wireless networks, e.g., unreliable or lossy channels, limited and unpredictable throughput, *etc*. These characteristics of wireless networks and wireless devices impose additional requirements when it comes to implementing data science models in a real system. Consequently, the key implementation challenges will depend on whether the data-driven prediction task had to be executed on the wireless node or the wireless network. Basically, two system models can be anticipated: Local models and Global models. Whether a local or a global model has to be deployed will depend on the target scenario and wireless problem.

*Local models* Local models are models deployed directly on the wireless nodes. The key implementation challenges in this deployment scenario are related to the available processing power and energy resources on the wireless nodes. All data processing and calculations for extracting knowledge, *i.e*., data pre-processing and predicting the accurate hypothesis need to be carried out locally with regard to the available resources. In order to adjust to these limitations, a trade-off between model complexity and model accuracy should be considered. Model complexity refers to the computational/processing and power consumption requirements on the hardware. The higher the required model accuracy, the higher the computational and energy consumption requirements are. In case the required accuracy or the model complexity cannot be satisfied by the node’s resources, the prediction task may be offloaded to a more powerful centralized point in the network. However, this comes at the cost of additional power consumption and network overhead as discussed in the next paragraph.

*Global models* Global models are deployed at a central point in the network: a more powerful wireless node, a dedicated server, or a dedicated cloud computing infrastructure. A global model can execute a prediction task after obtaining a global view of the network using the information from all wireless nodes that are part of the considered scenario. The key implementation challenges of a global model are related to the constraints of the wireless network (e.g., available bandwidth), as well as on the available processing and power resources of the local nodes, as follows:

1. *Transmission power consumption:* Compared to the local model where the main limitation factor for model implementation is the computing power consumption (*i.e*., power consumption due to heavy computations), in a global model deployment the processing tasks at the nodes need to be optimized to reduce the transmitting power consumption (*i.e*., power consumption due to extensive transmission of information to the central node).
2. *Network throughput consumption:* A global model makes predictions based on data that is being sampled by local nodes and sent over the wireless network to the target central point having the global model deployed. Forwarding each particular data sample, also called raw data, may result in high bandwidth consumption as well as transmission power consumption on the local nodes. One way to prevent unnecessary communication overhead is to perform data pre-processing already at the nodes locally, and send only aggregate reports to the central point. Advanced deployments may consider also to distribute the processing load related to the data mining task over several wireless nodes. In this way, each local node will contains partial event patterns and transmit only reduced data amounts with partial mining results to the central point. One example can be found in an earlier introduced work [99].
3. *Large data volumes:* Even after applying data aggregation and distributed data mining techniques to reduce data transmissions, large-scale wireless networks with thousands of nodes may produce large volumes of data due to continuous transmissions by heterogeneous devices. The performance for processing and mining the data samples is limited by the central point’s hardware resources, which are too expensive to be updated frequently. Several parallel programming models have been introduced to process large amounts of data in a fast and efficient way using distributed processing across clusters of computers. There is still open research in adapting some data mining algorithms for these parallel programming platforms (e.g., parallel k-means clustering).

## 5. Conclusions

Due to the recent successes of wireless testing facilities, more and more wireless data can be obtained from online repositories and/or wireless testbed facilities. In response to the need for efficient knowledge extraction from these large databases and large datasets, data mining has become a rapidly developing technology for which specific standards are established over the past few years. During this process it has evolved into a mature research and technology area becoming the heart of the broader Knowledge Discovery Process and data science in general. As a result, data science has found several applications in the domain of wireless communication and networks.

However, due to their very specialized expertises, the research gap between wireless experts and experienced data science experts is still quite large. To remedy this situation, this paper gave an introduction about the use of data science in the context of wireless networks. A high level overview was given about the categories of wireless problems in which data science techniques can be applied and the uitability of a number of frequently used learning algorithms were discussed in the context of wireless research problems. To further demonstrate the benefits of data science, an overview was given of existing data-driven wireless research papers and the role of data science in these publications was further discussed.

Although an increasing number of research papers is based on data-driven research, not all of these research papers utilize a methodology that fully complies with standard approaches developed and accepted by the data science community. To remedy this, the paper introduced a high level framework for knowledge extraction consisting of a 6-step methodology, and illustrated the methodology with wireless network research examples. It is important to note that data mining is just one step of the overall knowledge discovery process, but that additional steps such as problem definition, data analysis, pre-processing and performance analysis are an equally important part to successfully pursue a data-driven approach for knowledge discovery in wireless networks.

To further assist wireless engineers in the understanding of the concepts of data science and to show fruitful ways of pursuing their research during each knowledge discovery step, the methodology was then applied in the form of a wireless data science tutorial case study using an extensive real-world dataset to identify devices and device types based on their traffic patterns. However, note that the presented techniques, algorithms and the overall methodology are generic, as such are not limited to the particular problem use case. In this tutorial, different knowledge discovery steps are re-evaluated by considering real-life examples and examples are given for troubleshooting potential problems in the context of wireless network research. All datasets and scripts used during the tutorial are available online for interested researchers and can be used for teaching or other educational purposes. Dataset: [24] and Scripts: [23].

In summary, the main purpose of this paper was to help engineers understand the concepts of data science by explaining various aspects and importance of each knowledge discovery step in detail, starting from a high level overview and theoretical concepts and then referring to practical examples. Providing such an overview and tutorial at this moment, at a time when more and more wireless data can be found in online repositories and/or is generated by existing wireless deployments should have a positive impact in guiding wireless researchers and their work.

## Figures and Tables

**Table 1:** Summary of types of learning paradigms.

	Categorization Criteria	Learning Types	Comment
**Learning paradigms**	Amount of feedback given to the learner	Supervised	The learner knows all inputs/outputs
		Unsupervised	The learner knows only the inputs
		Semi-supervised	The learner knows only a few input/output pairs
	Amount of information given to the learner	Offline	The learner is trained on the entire dataset
		Online	The learner is trained sequentially as data becomes available
		Active	The learner selects the most useful training data

**Table 2:** Summary of various data science applications in wireless networks.

*Problem Type*	Optimizing Wireless Network Performance				Information Processing for Wireless Network Applications		
	MAC	Routing	Data Aggregation	Cognitive Radio	Activity Recognition	Security	Localization
Regression		[94]					[33]
Classification	[29]	[9,10]	[99]	[36,37,97,107]	[109]		
Clustering			[74]				
Anomaly Detection						[42,87]	
Summarization			[31]				

**Table 3:** Isolated testbed data summary describing (i) the device types; (ii) the number of devices for each of these types; (iii) the traffic type and number of data traces for each traffic type and (iv) minimum, maximum, average and standard deviation of the number of data points per data trace.

Device Type	Number of Devices	Traffic Type	Minimum	Maximum	Average	Standard Deviation
Dell Netbook	5	iPerf TCP (1 case × 5 traces)	841,299	3,059,247	1,820,062	948,900
		iPerf UDP (3 cases × 5 traces)	298,956	5,702,776	2,382,538	1,799,493
		Ping ICMP (2 cases × 3 traces)	359,220	359,996	359,865	316
		SCP TCP (1 case × 5 traces)	1,514,216	1,569,352	1,543,571	27,750
iPad	3	iPerf TCP (1 case × 3 traces)	1,305,673	1,780,640	1,527,179	239,090
		iPerf UDP (3 cases × 3 traces)	297,957	2,181,618	1,305,483	769,987
		Ping ICMP (2 cases × 3 traces)	301,966	322,124	309,749	7,991
		SCP TCP (1 case × 3 traces)	1,598,030	1,847,037	1,749,059	132,710
iPhone	4	iPerf TCP (1 case × 4 traces)	440,623	4,162,438	2,357,540	2,072,695
		iPerf UDP (3 cases × 4 traces)	306,413	4,094,728	1,791,755	1,378,019
		Ping ICMP (2 cases × 4 traces)	314,176	673,590	494,099	190,049
		SCP TCP (1 case × 4 traces)	599,460	1,599,098	1,348,888	499,619
Nokia Phone	2	iPerf TCP (1 case × 2 traces)	718,480	844,531	781,505	89,131
		iPerf UDP (3 cases × 2 traces)	300,924	5,131,699	2,189,739	2,094,815
		Ping ICMP (2 cases × 2 traces)	250,532	359,209	331,915	54,255
		SCP TCP (1 case × 2 traces)	1,316,782	1,570,745	1,443,763	179,579

**Table 4:** 5 number summary of the TCP inter-arrival times (IAT) data.

Device Type	Device	min[$\times 10^{-6}$][s]	Q1[$\times 10^{-4}$][s]	median[$\times 10^{-4}$][s]	Q3[$\times 10^{-4}$][s]	max[s]
Dell Netbook	DN1	5.96	6.91	8.24	13	0.1872
	DN2	5.96	5.67	6.20	7.35	0.2845
	DN3	5.96	5.72	6.18	7.70	0.1769
	DN4	2.86	4.16	5.80	6.56	0.2017
	DN5	2.86	3.82	4.72	6.22	0.6571
iPad	iPad1	1.91	5.46	6.20	12	5.2795
	iPad2	1.91	5.80	7.83	13	10.3603
	iPad3	2.86	16	19	34	0.1102
iPhone 3G and 4G	iPhone3G1	2.86	6.84	9.82	21	2.95
	iPhone3G2	1.91	15	18	35	0.4726
	iPhone4G1	3.81	9.55	13	25	0.1456
	iPhone4G2	4.77	14	16	31	0.1487
Nokia Phone	NP1	1.91	5.39	6.43	11	0.1481
	NP2	1.91	5.50	6.49	11	0.1486

**Table 5:** $R^{2}$ values for 50,000 bins histograms for iPerf TCP IATs for the traces from the 14 devices described in Table 3.

	DN1	DN2	DN3	DN4	DN5	iPad1	iPad2	iPad3	iPhone1	iPhone2	iPhone3	iPhone4	Nokia1	Nokia2
DN1	1	0.2407	0.2213	0.1624	0.0968	0.1130	0.1326	0.0527	0.3364	0.0770	0.1858	0.0412	0.2390	0.1935
DN2	0.2407	1	0.9981	0.8620	0.6961	0.9248	0.7738	0.0656	0.4497	0.1512	0.2688	0.0133	0.8659	0.8382
DN3	0.2213	0.9981	1	0.8604	0.6950	0.9273	0.7702	0.0657	0.4633	0.1508	0.2694	0.0122	0.8656	0.8391
DN4	0.1624	0.8620	0.8604	1	0.9581	0.8056	0.6613	0.0616	0.4238	0.1213	0.2278	0.0106	0.8212	0.7756
DN5	0.0968	0.6961	0.6950	0.9581	1	0.6550	0.5230	0.0493	0.3408	0.0901	0.1667	0.0057	0.6870	0.6396
iPad1	0.1130	0.9248	0.9273	0.8056	0.6550	1	0.9285	0.0818	0.3830	0.1646	0.3298	0.0626	0.8741	0.8724
iPad2	0.1326	0.7738	0.7702	0.6613	0.5230	0.9285	1	0.1207	0.3734	0.2001	0.4456	0.1948	0.8286	0.8337
iPad3	0.0527	0.0656	0.0657	0.0616	0.0493	0.0818	0.1207	1	0.1575	0.6292	0.2366	0.1978	0.1028	0.0986
iPhone1	0.3364	0.4497	0.4633	0.4238	0.3408	0.3830	0.3734	0.1575	1	0.1709	0.3572	0.0592	0.5170	0.4687
iPhone2	0.0770	0.1512	0.1508	0.1213	0.0901	0.1646	0.2001	0.6292	0.1709	1	0.2353	0.4011	0.1837	0.1778
iPhone3	0.1858	0.2688	0.2694	0.2278	0.1667	0.3298	0.4456	0.2366	0.3572	0.2353	1	0.1486	0.5019	0.5197
iPhone4	0.0412	0.0133	0.0122	0.0106	0.0057	0.0626	0.1948	0.1978	0.0592	0.4011	0.1486	1	0.0460	0.0411
Nokia1	0.2398	0.8659	0.8656	0.8212	0.6870	0.8741	0.8286	0.1028	0.5170	0.1837	0.5019	0.0460	1	0.9922
Nokia2	0.1935	0.8382	0.8391	0.7756	0.6396	0.8724	0.8337	0.0986	0.4687	0.1778	0.5197	0.0411	0.9922	1

**Table 6:** $R^{2}$ values for 50.000 bin histograms for iPerf TCP IATs for traces concatenated by same device type.

	DN	iPad	iPhone	Nokia
DN	1	0.6254	0.4013	0.8235
iPad	0.6254	1	0.6562	0.7359
iPhone	0.4013	0.6562	1	0.5188
Nokia	0.8235	0.7359	0.5188	1

**Table 7:** Performance evaluation of *k*-NN trained on 500-bin histograms for device type classification for different values of data cleaning thresholds, and the corresponding data loss.

Threshold →	0.3	0.1	0.01
Misclassification error	5.3%	3.4%	0.8%
Average data loss	0.0032%	0.035%	0.387%

**Table 8:** $R^{2}$ values for 500 bin histograms for iPerf TCP IATs for the traces from five Dell Netbooks cleaned using Th=0.1.

	DN1	DN2	DN3	DN4	DN5
DN1	1	0.5915	0.6084	0.3660	0.1787
DN2	0.5915	1	0.9991	0.8546	0.6394
DN3	0.6084	0.9990	1	0.8516	0.6350
DN4	0.3660	0.8546	0.8516	1	0.9356
DN5	0.1787	0.6394	0.6350	0.9356	1

**Table 9:** Strategies for improving the performance of a learning algorithm. Adapted from [187]. The asterisk (*) denotes solutions that are not applicable to non-parametric learning algorithms such as *k* nearest neighbors (*k*-NN) and decision trees.

Diagnosed Problem	Solution
The model is suffering from high variance	Utilize more training data
	Try a smaller set of features
	Reduce the model complexity
	Increase regularization (* for parametric models)
The model is suffering from high bias	Reduce the number of training instances (also increase speed)
	Obtain additional features
	Increase the model complexity
	Decrease regularization (* for parametric models)
Convergence problem	Use more training iterations
	Reduce the learning rate (* for parametric models)

**Table 10:** *k*-NN performance for device classification.

*dell1*	*dell2*	*dell3*	*dell4*	*dell5*	*ipad1*	*ipad2*	*ipad3*	*iphone1*	*iphone2*	*iphone3*	*iphone4*	*nokia1*	*nokia2*	← predicted
164	3	0	0	0	1	0	0	0	0	0	0	0	0	*dell1*
0	190	61	7	0	0	1	0	0	0	0	0	5	1	*dell2*
0	36	217	3	0	0	0	0	0	0	0	0	0	1	*dell3*
0	3	1	472	34	0	0	0	0	0	0	0	4	2	*dell4*
0	0	2	31	576	0	0	0	0	0	0	0	2	0	*dell5*
0	44	18	5	0	58	37	0	0	0	0	0	4	2	*ipad1*
0	27	12	1	1	7	82	0	0	0	0	0	8	5	*ipad2*
0	0	0	0	0	0	0	299	0	0	0	0	0	0	*ipad3*
3	3	2	2	0	1	0	0	342	0	0	0	2	0	*iphone1*
0	0	0	0	0	0	0	3	0	257	0	0	0	0	*iphone2*
0	0	0	0	0	0	0	0	0	0	137	0	0	0	*iphone3*
0	0	0	0	0	0	0	0	0	2	0	86	0	0	*iphone4*
0	3	0	7	1	0	1	0	0	0	0	0	536	284	*nokia1*
0	0	1	1	0	0	0	0	0	0	0	0	208	617	*nokia2*

**Table 11:** Summarized classification performance for device type classification.

Algorithm	Confusion Matrix					*Precision*	*Recall*
	*Dell*	*Ipad*	*Iphone*	*Nokia*	← Predicted				
***k-NN***	1807	1	0	12	*dell*	0.935	0.993
	111	479	0	21	*ipad*	0.98	0.784
	8	9	824	1	*iphone*	1	0.979
	7	0	0	1653	*nokia*	0.98	0.996
			*weighted average [%] →*			**96.7**	**96.6**
***Decision trees***	1807	1	0	12	*dell*	0.99	1
	111	479	0	21	*ipad*	0.995	0.992
	8	9	824	1	*iphone*	0.998	0.993
	7	0	0	1653	*nokia*	0.997	0.998
			*weighted average [%] →*			**99.7**	**99.7**
***Logistic regression***	1807	1	0	12	*dell*	0.973	0.982
	111	479	0	21	*ipad*	0.916	0.84
	8	9	824	1	*iphone*	0.966	0.884
	7	0	0	1653	*nokia*	0.939	0.999
			*weighted average [%] →*			**95.3**	**95.3**
***Neural networks***	1807	1	0	12	*dell*	0.443	0.699
	111	479	0	21	*ipad*	0.6	0.005
	8	9	824	1	*iphone*	0.627	0.6
	7	0	0	1653	*nokia*	0.398	0.3
			*weighted average [%] →*			**47.9**	**46.2**

**Table 12:** Confusion matrix for neural networks using the optimized configuration settings, α=0.1, 500 epochs, HLsigmoid=6.

*dell*	*ipad*	*iphone*	*nokia*	← predicted
1820	0	0	0	*dell*
0	611	0	0	*ipad*
0	0	842	0	*iphone*
0	0	0	1660	*nokia*

**Table 13:** Comparative study of the proposed methodology in Section 3 *vs.* the custom approach in [12].

		I: Custom Approach [12]	II: Proposed Methodology Section 3	*Recommendations*.
**Understanding the problem domain**	Problem formulation	Stated classification problem**✓**	Stated classification problem**✓**	Use guidelines in Section 2
	Data collection	Collected Experimental data**✓**	Experimental data from Repository**✓**	Use *real* data
**Understanding the data**	Data validation	-	5 number summary**✓**	Use visual and
	Hypothesis validation	Visual techniques**✓**	Computational techniques**✓**	computational techniques
**Data pre-processing**	Data cleaning	-	*Th*-based cleaning**✓**	Ensure reliability of data
	Data reduction	Histograms	Histograms with optimized granularity**✓**	Find optimal data representation
	Data transformation	-	*min-max* normalization**✓**	Increase computational efficiency
**Data mining**		NN**✓**	NN, k-NN, LR, DT**✓**	Select and evaluate the most suitable algorithm**s**
**Performance evaluation**	Metrics	Accuracy, Recall	Precision, Recall**✓**	Exploit best
	Model selection	Custom approach	Cross-validation**✓**	practices from the
	Results representation	Partial results	Complete results—confusion matrix**✓**	data science community (Section 4.6)
